# Proteomic and Metabolomic Characterization of Metabolically Healthy Obesity: A Descriptive Study from a Swedish Cohort

**DOI:** 10.1155/2021/6616983

**Published:** 2021-10-06

**Authors:** J. Korduner, P. M. Nilsson, O. Melander, M. J. Gerl, G. Engström, E. Bachus, M. Magnusson, F. Ottosson

**Affiliations:** ^1^Department of Clinical Sciences, Lund University, Malmö, Sweden; ^2^Department of Internal Medicine, Skåne University Hospital, Malmö, Sweden; ^3^Lipotype GmbH, Dresden, Germany; ^4^Wallenberg Center for Molecular Medicine, Lund University, Lund, Sweden; ^5^Department of Cardiology, Skåne University Hospital, Malmö, Sweden; ^6^North-West University, Hypertension in Africa Research Team (HART), Potchefstroom, South Africa

## Abstract

**Method:**

Associations between different biomarkers (proteomics, lipidomics, and metabolomics) coupled to either MHO or metabolically unhealthy obese (MUO) individuals were analyzed through principal component analysis (PCA). Subjects were identified from a subsample of 416 obese individuals, selected from the Malmö Diet and Cancer study—Cardiovascular arm (MDCS-CV, *n* = 3,443). They were further divided into MHO (*n* = 143) and MUO (*n* = 273) defined by a history of hospitalization, or not, at baseline inclusion, and nonobese subjects (NOC, *n* = 3,027). Two distinctive principle components (PL2, PP5) were discovered with a significant difference and thus further investigated through their main loadings.

**Results:**

MHO individuals had a more metabolically favorable lipid and glucose profile than MUO subjects, that is, lower levels of traditional blood glucose and triglycerides, as well as a trend of lower metabolically unfavorable lipid biomarkers. PL2 (lipidomics, *p*=0.02) showed stronger associations of triacylglycerides with MUO, whereas phospholipids correlated with MHO. PP5 (proteomics, *p*=0.01) included interleukin-1 receptor antagonist (IL-1ra) and leptin with positive relations to MUO and galanin that correlated positively to MHO. The group differences in metabolite profiles were to a large extent explained by factors included in the metabolic syndrome.

**Conclusion:**

Compared to MUO individuals, corresponding MHO individuals present with a more favorable lipid metabolic profile, accompanied by a downregulation of potentially harmful proteomic biomarkers. This unique and extensive biomarker profiling presents novel data on potentially differentiating traits between these two obese phenotypes.

## 1. Introduction

Although obesity is a well-established risk factor for the development of endemic modern Western public health problems, including cardiovascular disease (CVD) and type 2 diabetes (DM2) [1], accumulating evidence is suggesting that there is a small proportion of individuals with excess weight (body mass index (BMI) ≥ 30 kg/m^2^) that seem to escape these aforementioned conditions—a concept known as Metabolically Healthy Obesity (MHO) [2, 3]. Along with this phenomenon, there has been a debate concerning the heterogeneity of obesity, and the negative consequences of excess fat seem to be more complex and individually patterned than previously thought [2, 4]. However, there is no doubt that obesity in the majority of cases represents a state of increased risk. Even in obese individuals defined as MHO that seem to experience less negative effect of their excess weight, increasing evidence is suggesting that this could be a transient state and will eventually in due time transform into its unhealthier counterparts—Metabolically Unhealthy Obesity (MUO) [5–7].

There exists no agreed definition of MHO, but most studies on this topic suggest that it should involve a lack of risk factors for the metabolic syndrome (MetS) [8]. In a recent paper [9], we defined MHO as obese individuals (BMI ≥ 30 kg/m^2^) who had never been hospitalized for a somatic disease before study baseline (at mean age of 56 years) and described the prognosis regarding incident CVD and mortality risk of MHO subjects compared to MUO and nonobese controls (NOC) in a Swedish cohort from the 1990s—the Malmö Diet and Cancer Study (MDCS; *n* = 28,098). Our findings suggested that MHO individuals had a significantly lower risk of total mortality and developing CVD during a 20-year follow-up period, compared to MUO individuals. Interestingly, no differences in prospective risks could be seen when comparing MHO to NOC individuals. Descriptive data from the study showed that MHO individuals presented with a less sedentary lifestyle, held a higher educational level, and displayed a more favorable glucose and lipid blood profile [9]. These descriptive findings were in line with earlier publications [10, 11], although our definition of MHO was novel and differed from previous ones [9].

There is still no clear explanation as to which factors contribute to the development of MHO contra MUO, but many theories exist. One common assumption is that a chronic inflammatory state, commonly associated with obesity, is downregulated in MHO individuals [12]. This in turn could be interpreted as influenced by less pronounced nonalcoholic fatty liver disease (NAFLD), determined by genetic factors and/or a diversity of the gut microbiota [4]. Other benign attributes that attract one's attention is the distribution patterns (peripheral vs. central obesity) [13] and the expandability of adipose tissue [14], as well as the glucose and triglyceride index [15, 16], but also specific biomarkers associated with obesity such as adiponectin [17] and neurotensin [18].

With this in mind, there is an urge to better understand the true benign nature of obesity presented in some selected individuals and the factors associated with it, to improve and individualize the treatment and care of individuals with excess body weight. From our recent paper [9], we concluded that a sedentary lifestyle and higher levels of blood glucose and lipids, combined with adverse lower socioeconomic conditions, contribute negatively to unhealthy obesity. Nonetheless, we would like to elucidate further on the descriptive profile of MHO individuals and thus analyze selected biomarkers associated with this specific phenotype.

Consequently, this observational study aimed to better characterize the metabolic profile of previously defined MHO individuals [9] by comparing plasma levels of metabolites (metabolomics and lipidomics) and circulating proteins (proteomics) between MHO, MUO, and NOC subgroups.

## 2. Materials and Methods

### 2.1. Subjects

A total of 28,098 individuals were selected (41% attendance rate) to participate in the baseline examination of the MDCS between 1991 and 1996, which included risk factor assessment through laboratory testing, physical examination, and a questionnaire. A detailed description of the inclusion criteria [9] and methodological aspects has been previously published [19,20]. In short, obese individuals (BMI ≥ 30 kg/m^2^) were selected from the subcohort MDCS-Cardio Vascular arm (MDCS-CV). This subcohort was derived from the original MDCS, when every other individual included in the baseline examination was reinvited during 1992–1994 to participate in MDCS-CV (*n* = 6,103). The primary aim was to study the epidemiology of carotid artery disease, when also laboratory analyses of additional fasting blood samples were carried out [21, 22].

**Figure 1:**
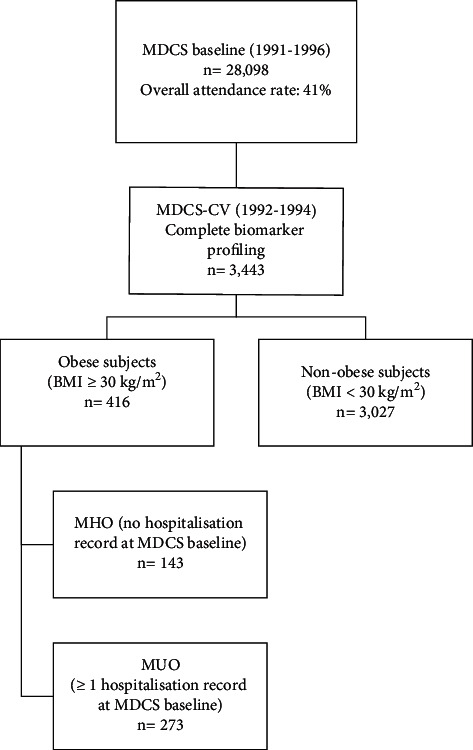
Flowchart of the MDCS-CV subcohort stratified for obese and nonobese subjects, respectively.

The number of included individuals was further reduced due to the lack of complete biomarker profiling data (*n* = 3,443). The obese individuals were then subdivided into two groups consisting of MHO and MUO, based upon the absence of hospitalization for somatic disease up until the inclusion at MDCS-baseline examinations (MHO) [9]. Hospitalization status was obtained through the Swedish National Hospital Inpatient Register, where external injuries/intoxications and normal deliveries were considered nonhospitalization and excluded. Furthermore, hospitalization status included only records for somatic disease. Obese individuals with no recorded history of hospitalization were considered MHO (*n* = 143), whereas MUO individuals were characterized as individuals with at least one record of hospitalization for somatic disease prior to inclusion at MDCS-baseline (*n* = 273). Moreover, selected MHO individuals were further compared with NOC subjects from the same subcohort (*n* = 3,027); see Figure 1 for a detailed flowchart. This novel approach of defining MHO individuals has been previously applied in the same cohort from an urban population [9].

### 2.2. Metabolite Profiling

Profiling of plasma metabolites was performed using a LC-QTOF-MS System (Agilent Technologies 1290 LC, 6550 MS, Santa Clara, CA, USA) and has previously been described in detail [23]. Briefly, overnight fasted citrate venous plasma samples stored at −80°C were thawed and extracted by addition of 120 *μ*l extraction solution (80 : 20 methanol/water) to 20 *μ*l plasma. The samples were then incubated at 4°C for 1 hour at 1250 rpm. After 15 min centrifugation at 14 000 g, 100 *μ*l supernatant was transferred into a glass vial for analysis. Extracted samples were separated on an Acquity UPLC BEH Amide column (1.7 *μ*m, 2.1 *∗* 100 mm; Waters Corporation, Milford, MA, USA). Metabolite identification, quality control, and normalization were performed as described previously [24].

### 2.3. Lipid Profiling

Lipid extraction of 1 *μ*L of overnight fasted citrate plasma samples was stored at −80°C upon collection, followed by quantitative mass spectrometry-based lipid analysis. The analysis was performed at Lipotype GmbH using a high-throughput shotgun lipidomics technology [25]. Lipid identifiers of the SwissLipids database [26] (https://www.swisslipids.org) are provided in Table S1.

### 2.4. Protein Profiling

Fasting plasma levels of 136 proteins were measured using Olink Proseek Multiplex proximity extension assay (PEA) at the Clinical Biomarkers Facility, Science for Life Laboratory, Uppsala, Sweden. PEA uses two oligonucleotide-labelled antibodies per protein, which form a PCR reporter sequence when both antibodies are bound to the target protein. The reported sequence is quantified by real-time quantitative polymerase chain reaction [27].

### 2.5. Statistical Analysis

Prior to statistical analysis, missing values for all biomarkers (maximum 20% missing allowed) were imputed using the NIPALS algorithm and were subsequently mean-centered and unit-variance scaled. Unsupervised dimension-reduction of each set of biomarker layers (metabolites, lipids, and proteins) was performed using principal component analysis (PCA). PCA was first performed for each biomarker layer in all obese participants and subsequently in all non-MUO participants in the same manner. For each biomarker layer, five principal components (PC) were calculated. In the participants with obesity, logistic regression models, adjusted for age and sex, were used to find associations between PCs and MHO (compared to MUO). PCs that were significantly associated with MHO were investigated for correlations with cardiometabolic risk factors using partial Spearman's correlation tests, adjusted for age and sex. Subsequent analysis in all non-MUO participants used logistic regression models to find associations between PCs and MHO (compared to NOC). All statistical analyses were performed in R 3.6.1. PCA and imputation were performed in the mixOmics [28] package and the partial Spearman's correlation tests in the ppcor [29] package. A *p* value <0.05 was considered significant.

## 3. Results

We observed differences in several cardiometabolic risk factors between subjects with MHO subjects compared to MUO (Table 1). MHO participants had a more favorable cardiometabolic risk factor profile compared to MUO, including lower BMI and waist circumference, proportion of prescribed antihypertensive drugs, and fasting levels of glucose and triglycerides, as well as higher levels of HDL cholesterol. Apart from lower BMI, NOC participants were characterized by lower waist circumference, systolic and diastolic blood pressure HbA_1c_, proportion of antihypertensive drugs, and fasting levels of glucose, triglycerides, and LDL cholesterol, but higher levels of HDL cholesterol.

**Figure 2:**
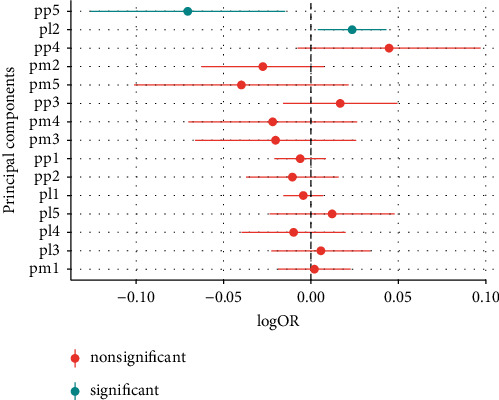
Logistic regression models, with significance testing, of the main PCs when comparing MHO (a) with MUO (b) subjects.

**Figure 3:**
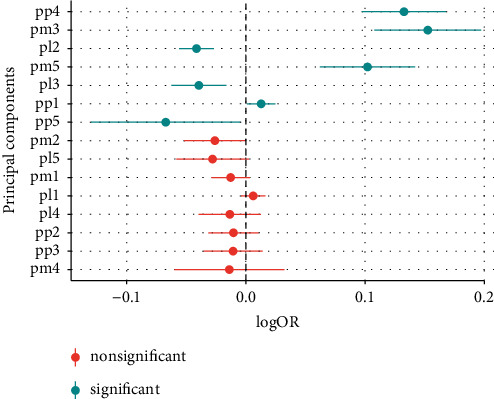
Main loadings for PL2, when comparing MHO with MUO.

**Figure 4:**
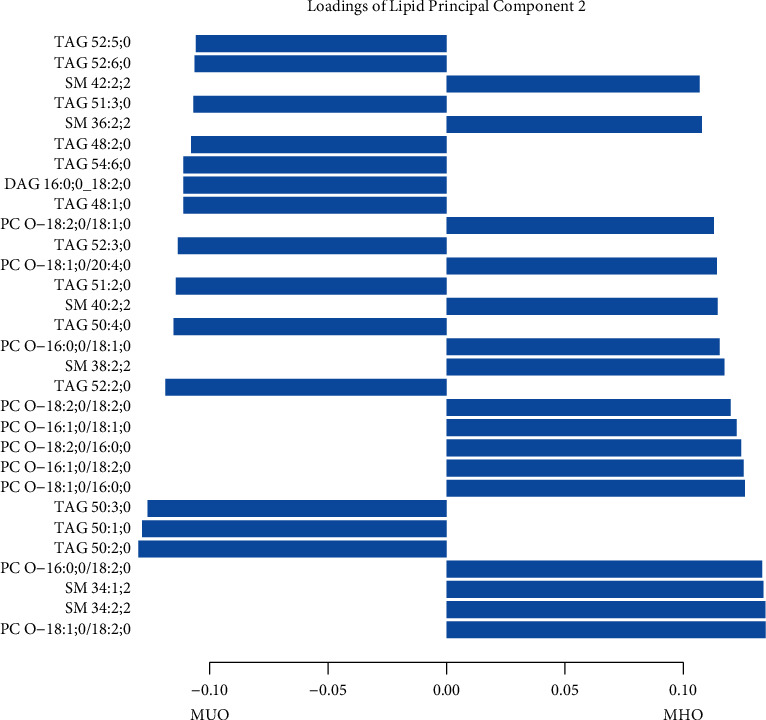
Main loadings for PP5, when comparing MHO with MUO.

Biomarker profiles were constructed in participants with obesity (BMI > 30 kg/m^2^), using PCA of three different biomarker layers, including either 112 metabolites, 184 lipids, or 136 proteins. The first five principal components (PC) in each biomarker layer could explain 41.8% of the metabolite variation, 63.8% of the lipid variation, and 53.1% of the protein variation, respectively (Table S2). To investigate whether the obesity biomarker patterns were related to MHO, all 15 biomarker PCs were analyzed using logistic regression models. The second lipid PC (PL2) (odds ratio, OR 1.06, *p*=0.018) and the fifth protein PC (PP5) (OR 0.85, *p*=0.013) were associated with MHO (Figure 2). PL2 was dominated by positive contributions from phospholipids, such as sphingomyelins and phosphatidylcholine ethers, and negative contributions from triacylglycerides (Figure 3). The strongest positive contribution to PP5 was interleukin-1 receptor antagonist (IL1-RA) followed by leptin and fatty acid-binding protein 4, while the strongest negative contributions were from galanin (Figure 4). Loadings for all PCs are presented in Tables S3–S5.

**Figure 5:**
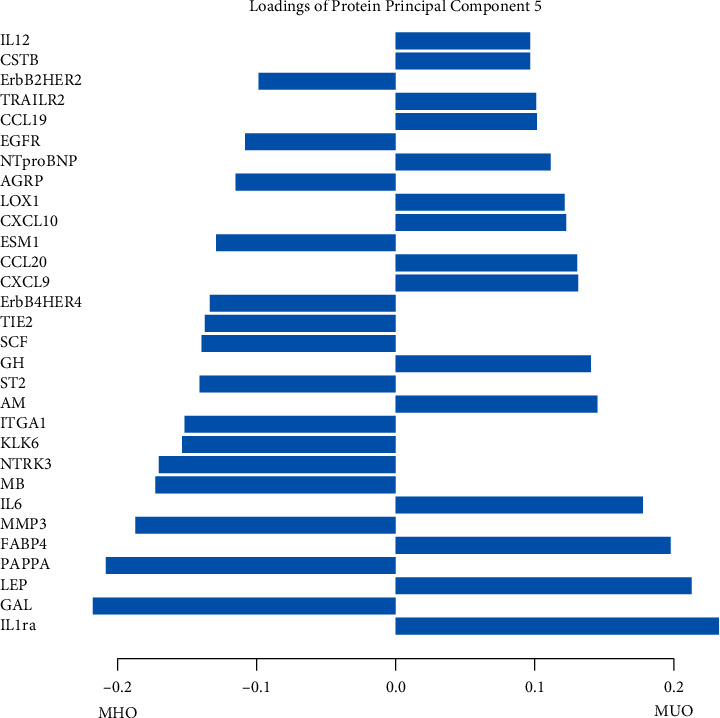
Correlation between biomarker principal components and cardiometabolic risk factors. Correlations between cardiometabolic risk factors and lipid principal component 2 (pl2) and protein principal component 5 (pp5) are expressed as partial Spearman's correlation coefficients, adjusted for age and sex. AHT: antihypertensive treatment; CRP: C-reactive protein; DBP: diastolic blood pressure; SBP: systolic blood pressure; HbA1c: glycated haemoglobin; HOMA-IR: homeostatic model assessment for insulin resistance.

Both MHO-associated PCs were correlated with traditional cardiometabolic risk factors (Figure 5, Table S6). PL2 showed strong inverse correlations with plasma triglycerides (rho = −0.67, *p* < 0.001), HOMA-IR (rho = −0.36, *p* < 0.001), and glucose (rho = −0.32, *p* < 0.001) and strong positive correlations with HDL cholesterol (rho = 0.59, *p* < 0.001). PP5 was strongly correlated with CRP (rho = 0.36, *p* < 0.001) and waist circumference (rho = 0.26, *p* < 0.001) but inversely correlated with HDL cholesterol (rho = −0.27, *p* < 0.001). All correlations between MHO-related PC and cardiometabolic risk factors are depicted in Figure 5.

When adjusting for combined components of the MetS, according to National Cholesterol Education Program panel III (NCEP III) criteria [30] (systolic blood pressure, plasma glucose, HDL cholesterol, triglycerides, and waist circumference), no significant differences could be seen between the PCs. Strong contributors to differences in PL2 were HDL cholesterol and triacylglycerides (Table 2).

**Figure 6:**
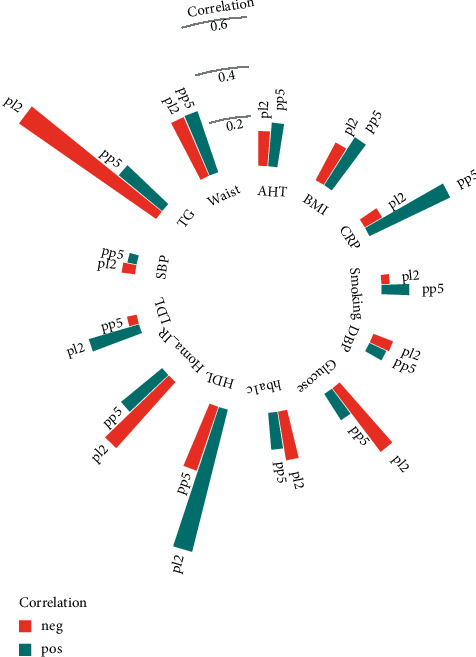
Logistic regression models, with significance testing, of the main PCs when comparing MHO (a) with NOC subjects (b).

PCA of three different biomarker layers was used to describe the biomarker variation of study participants without MUO. The first five principal components (PC) in each biomarker layer could explain 43.0% of the metabolite variation, 53.5% of the lipid variation, and 61.4% of the protein variation (Table S2). In general, there were larger differences in the biomarker pattern between NOC and MHO subjects, than between MHO and MUO subjects. Seven PCs, three protein PCs, two lipid PCs, and two metabolite PCs were associated with increased odds of MHO as compared to NOC (Figure 6). Similar to PP4 in the obese individuals, PP5, which was the PC that was most strongly associated with increased odds of MHO over NOC, had strong positive contributions from IL1-RA and IL6, but negative contributions from galanin and pappalysin-1. The lipid PC, showing the largest differences between MHO and NOC, PL2, was dominated by negative contributions from triacylglycerides and positive contributions from phosphatidylcholine ethers, similar to PL2 in the obese population (Tables S7–S9).

## 4. Discussion

This observational study from an urban population ran an extensive biomarker profiling of 432 lipids, metabolites, and proteins across two distinct obese subgroups, MHO and MUO—an unexplored field as of yet. Key biomarker pattern findings include additional evidence of MHO individuals holding a more metabolically favorable lipid and glucose profile, that is, lower levels of traditional blood glucose and triglycerides, as well as a trend of lower levels of metabolically unfavorable lipid biomarkers. Even if significance levels were modest, PCA discovered nominally significant proteomic and lipidomic biomarkers that differed between the MHO and MUO subgroups. These differences were to a large extent explained by factors related to the MetS. When comparing MHO individuals to NOC, PCA of selected biomarkers and descriptive data demonstrated expected findings of obesity-related parameters.

### 4.1. Lipidomics

Lipidomic patterns display MHO-related principal component (PL2) with negative contributions of triacylglycerides and diacylglycerides when compared to MUO. This supports the notion of a more benign lipid profile of MHO subjects, since higher levels of triglycerides mirror a more metabolically active adipose tissue as well as atherogenic properties, thus with the MetS [31]. Furthermore, glycerphospholipids (exclusively ether phosphatidylcholine) and sphingomyelins seem to be associated positively with the MHO-related PL2. It is unclear whether phospholipids contribute positively or negatively to cardiovascular disease and metabolic disorders [32, 33]. However, research shows that ether phosphatidylcholine with shorter fatty acids and smaller amounts of total double bonds (more positively correlated with MHO) is increased in long-lived humans [34]—suggesting that perhaps MHO individuals deal better with oxygen stress than their counterparts, a theory supported by a previously cited systematic review, revealing that plasmalogens present a negative correlation with obesity, DM2, prediabetes, and CVD—all conditions associated with elevated levels of oxidative stress [32]. Moreover, sphingolipids (mainly sphingomyelin) seem to contribute to adipose tissue inflammation and the accompanying liver steatosis and insulin resistance; that is why their positive relationship with MHO appears more sophisticated than expected [32, 35]. The finding should be interpreted with caution given the relatively modest level of significance and number of PCs analyzed.

### 4.2. Proteomics

#### 4.2.1. Interleukin-1 Receptor Antagonist (IL-1ra)

Our study presented positive contributions of IL-1ra with the MUO-related PP5, compared to MHO. It has been debated whether IL-1ra is benign or if elevated levels of this biomarker present with adverse effects [36]. Indeed, it works as an inhibitor of the well-known proinflammatory cytokine interleukin-1*β* (IL-1*β*), involved in the development of various chronic inflammatory disorders, as well as CVD and DM2 [37]. The randomized double-blind CANTOS study contributed to the increasing evidence of positive effects of IL-1ra focused treatment, displaying that anti-inflammatory targeting with monoclonal antibodies against IL-1*β* significantly reduced recurrent cardiovascular events compared to placebo in postmyocardial infarction survivors [38]. However, conflicting data postulates that this protein is upregulated as a protective response to increased activities of IL-1*β* and interleukin-1*α* (IL-1*α*) [39]. Furthermore, additional findings hypothesize that IL-1RA might have harmful cardiovascular effects of its own and additionally prevent potentially positive effects of IL-1*α* and IL-1*β* [36].

#### 4.2.2. Galanin

An obesity-related neuropeptide was found negatively related to the MUO-correlated PP5, suggesting a relationship with MHO. Galanin is mainly involved in energy homeostasis, where increased hormone levels contribute to the development of obesity, through orexigenic effects, and also obesity-associated metabolic impairments, regardless of feeding regulation [40, 41]. Nevertheless, one study reports potential positive effects of this hormone, where it seems to improve glucose metabolism and uptake, thus decreasing insulin resistance [42].

#### 4.2.3. Leptin

Being a well-known biomarker for obesity, leptin correlated positively with MUO-related PP5. This adipocyte-derived hormone, increases with BMI and adipose tissue mass, suggesting that obese individuals develop an insensitivity to this hormone with increasing weight [43]. The hormone regulates the energy balance by inhibiting hunger mediated through the hypothalamus; hence, it works to reduce caloric intake and increase energy expenditure [44], suggesting that such obesity-promoting mechanisms might be more pronounced in MUO than in MHO.

### 4.3. Study Limitations

This is the first study of its kind, including 432 metabolites and proteins aiming to describe their relationship with metabolically healthy versus unhealthy obesity. Still, several limitations should be considered in this study. MDCS, although being a well-characterized, population-based prospective cohort with a large number of included individuals, had a relatively poor overall attendance rate (41%) which could imply a health selection bias. Furthermore, both at baseline examination and reflected in our study sample, there exists a gender imbalance with a predominance of women.

A major limitation is the small sample size, resulting in limited power. Moreover, although we applied a data-reducing strategy, several derived PCs were tested with obesity phenotypes with the risk of false-positive results. This underlines the need for replication of the reported findings.

When performing a multiple regression analysis, adjusting for the components of the MetS [30], the associations of the biomarker PCs when comparing MHO and MUO individuals were attenuated (Table 2). This suggests that the difference of biomarker variation between MHO and MUO subjects in part could be explained by the MetS. Thus, one might argue that to keep the MHO phenotypic state and avoid hospitalization, there should be an ambition of the individual for weight stability and keeping a healthy lifestyle to avoid transformation into MUO linked to the MetS.

## 5. Conclusion

We have performed a plasma metabolic and protein profiling of MHO and MUO individuals, defined by absence (MHO) or presence (MUO) of a history of hospitalization for a somatic disease until midlife. Despite relatively weak associations, this novel approach confirms that MHO individuals present with a positive association with phosphatidylcholine ethers and sphingomyelins, as well as negative associations with triacyl- and diacylglycerides compared to MUO subjects. Furthermore, MHO individuals are characterized by the downregulation of potentially harmful proteomic biomarkers, compared to their MUO counterparts. A large part of the difference could be explained by the influence of MetS. Our research is in line with previous findings, although a unique and extensive biomarker profiling presents novel data on potential differentiating traits between these two obese phenotypes.

## Figures and Tables

**Table 1:** Descriptive comparison and significance testing for MHO (*n* = 143) compared to MUO subjects (*n* = 273) and MHO compared to NOC subjects (*n* = 3,027), with standard deviation (SD) or percentage (%) for metric and categorical variables, respectively.

Variable	MHO (*N* = 143)	MUO (*N* = 273)	*p*	NC (*N* = 3027)	*p*
Age (years)	57.7 (5.7)	59.1 (5.9)	**0.02**	57.4 (6.0)	0.50
Sex (% women)	65.7	65.6	0.97	59.2	0.11
BMI (kg/m^2^)	32.2 (2.3)	33.4 (3.34)	**<0.001**	24.6 (2.8)	**<0.001**
Waist (cm)	97.0 (12)	99.5 (12)	**0.04**	80.9 (11)	**<0.001**
SBP (mmHg)	150 (19)	148 (19)	0.51	140 (19)	**<0.001**
DBP (mmHg)	90.9 (9.7)	91.0 (9.4)	0.90	86.1 (9.2)	**<0.001**
Smoker (%)	21.1	15.8	0.19	27.6	0.07
AHT drug (%)	19.6	37.7	**<0.001**	14.1	0.11
Glucose (mmol/L)	5.53 (1.4)	5.90 (1.9)	**0.03**	5.09 (1.2)	**<0.001**
HbA_1c_ (%)	5.11 (0.82)	5.29 (1.0)	0.054	4.88 (0.68)	**0.001**
HOMA-IR	3.66 (7.5)	3.81 (4.0)	0.82	1.67 (1.9)	**0.002**
TG (mmol/L)	1.57 (0.72)	1.75 (0.80)	**0.02**	1.25 (0.60)	**<0.001**
HDL-C (mmol/L)	1.27 (0.32)	1.20 (0.28)	**0.03**	1.43 (0.38)	**<0.001**
LDL-C (mmol/L)	4.35 (1.1)	4.35 (1.1)	0.99	4.13 (0.97)	**0.02**
CRP (mg/L)	0.39 (0.50)	0.41 (0.44)	0.74	0.23 (0.40)	**<0.001**
MetS (%)	49.7	61.5	**0.03**	13.6	**<0.001**

AHT: antihypertensive treatment; DBP: diastolic blood pressure; SBP: systolic blood pressure: HbA1c: glycated haemoglobin; HOMA-IR: homeostatic model assessment for insulin resistance; CRP: C-reactive protein; MetS: metabolic syndrome.

**Table 2:** Multiple regression model (linear logistic regression) displaying odds ratios which indicate associations between biomarker principal components and MHO, compared to MUO.

Model	PP5		PL2	
	*p*	OR	*p*	OR
Model 1 (age + sex)	0.01^*∗*^	0.85	0.02^*∗*^	1.06
Model 1 + systolic blood pressure	0.01^*∗*^	0.85	0.02^*∗*^	1.06
Model 1 + plasma glucose	0.02^*∗*^	0.86	0.06	1.05
Model 1 + HDL cholesterol	0.06	0.88	0.35	1.03
Model 1 + triglycerides	0.04	0.87	0.24	1.04
Model 1 + waist circumference	0.07	0.91	0.08	1.04
Model 1 + metabolic syndrome	0.17	0.91	0.91	1.00

Odds ratios (OR) indicate associations between biomarker principal components and MHO (=1), compared to MUO (=0). Model 1 was adjusted for age and sex. PL2: lipidomic principal component 2. PP5: proteomic principal component 5. The model adjusted for metabolic syndrome was adjusted for all factors of the metabolic syndrome according to National Cholesterol Education Program panel III (NCEP III) criteria [30] (systolic blood pressure, fasting plasma glucose, HDL cholesterol, triglycerides, and waist circumference). *∗*Significant at *p* < 0.05.

## Data Availability

All requests for data access should be addressed to the corresponding author. Proposals requesting data access will have to specify how they plan to use the data.

## Abbreviations

CVD: Cardiovascular disease
CRP: C-reactive protein
DM2: Type 2 diabetes
FABP4: Fatty acid-binding protein 4
HOMA-IR: Homeostatic model assessment for insulin resistance
IL-1RA: Interleukin-1 receptor antagonist
MDCS: Malmö diet cancer study
MDCS-CV: Malmö diet cancer study—cardiovascular arm
MHO: Metabolically Healthy Obesity
MUO: Metabolically unhealthy obesity
NAFLD: Nonalcoholic fatty liver disease
NOC: Nonobese controls
PAPPA: Pappalysin-1
PC: Principal component
PCA: Principal component analysis.

## Supplementary Materials

Supplementary Materials Supplementary tables (S1–S9): index and contain additional data as follows: S1: table of SwissLipids IDs. S2: proportion of explained variance for the first 5 PCs in each biomarker layer (metabolite, lipid, and protein). The proportion of explained variance is presented for the principal component analysis performed for the obese participants (*n* = 416) (MHO + MUO) and the non-MUO (MUO + NOC) (*n* = 3,027). S3: loadings of metabolite PCs 1–5 (MHO vs. MUO). S4: loadings of lipid PCs 1–5 (MHO vs. MUO). S5: loadings of protein PCs 1–5 (MHO vs. MUO). S6: correlation between biomarker PCs (PL2, PP5) and cardiometabolic risk factors. S7: loadings of metabolite PCs 1–5 (MHO vs. NOC). S8: loadings of lipid PCs 1–5 (MHO vs. NOC). S9: loadings of protein PCs 1–5 (MHO vs. NOC).
