# Supplementary material for: Proteomic and Metabolomic Characterization of Metabolically Healthy Obesity: A Descriptive Study from a Swedish Cohort
Source: J Obes. 2021 Oct 6;2021:6616983. doi: 10.1155/2021/6616983 (PMC8514926; doi:10.1155/2021/6616983)
Supplement: Supplementary Materials — Supplementary tables (S1–S9): index and contain additional data as follows: S1: table of SwissLipids IDs. S2: proportion of explained variance for the first 5 PCs in each biomarker layer (metabolite, lipid, and protein). The proportion of explained variance is presented for the principal component analysis performed for the obese participants (n = 416) (MHO + MUO) and the non-MUO (MUO + NOC) (n = 3,027). S3: loadings of metabolite PCs 1–5 (MHO vs. MUO). S4: loadings of lipid PCs 1–5 (MHO vs. MUO). S5: loadings of protein PCs 1–5 (MHO vs. MUO). S6: correlation between biomarker PCs (PL2, PP5) and cardiometabolic risk factors. S7: loadings of metabolite PCs 1–5 (MHO vs. NOC). S8: loadings of lipid PCs 1–5 (MHO vs. NOC). S9: loadings of protein PCs 1–5 (MHO vs. NOC). [file 6616983.f1.docx]

**Table S1.** Table of SwissLipids IDs.

| **SwissLipids Name** | **SwissLipids ID** |
| --- | --- |
| Sterol ester (27:1/14:0) | SLM:000500342 |
| Sterol ester (27:1/15:0) | SLM:000500343 |
| Sterol ester (27:1/16:0) | SLM:000500346 |
| Sterol ester (27:1/16:1) | SLM:000500345 |
| Sterol ester (27:1/17:0) | SLM:000500347 |
| Sterol ester (27:1/17:1) |  |
| Sterol ester (27:1/18:0) | SLM:000500352 |
| Sterol ester (27:1/18:1) | SLM:000500351 |
| Sterol ester (27:1/18:2) | SLM:000500350 |
| Sterol ester (27:1/18:3) | SLM:000500349 |
| Sterol ester (27:1/20:2) | SLM:000500357 |
| Sterol ester (27:1/20:3) | SLM:000500356 |
| Sterol ester (27:1/20:4) | SLM:000500355 |
| Sterol ester (27:1/20:5) | SLM:000500354 |
| Sterol ester (27:1/22:6) | SLM:000500361 |
| Ceramide (d40:1) | SLM:000391319 |
| Ceramide (d40:2) | SLM:000391317 |
| Ceramide (d42:1) | SLM:000391346 |
| Ceramide (d42:2) | SLM:000391345 |
| cholesterol | SLM:000000287 |
| Diacylglycerol (16:0_18:1) | SLM:000308862 |
| Diacylglycerol (16:0_18:2) | SLM:000308863 |
| Diacylglycerol (16:1_18:1) | SLM:000308894 |
| Diacylglycerol (18:1_18:1) | SLM:000309012 |
| Diacylglycerol (18:1_18:2) | SLM:000309013 |
| Diacylglycerol (18:1_18:3) | SLM:000309014 |
| Phosphatidylcholine (14:0_0:0) | SLM:000063555 |
| Phosphatidylcholine (16:0_0:0) | SLM:000063723 |
| Phosphatidylcholine (16:1_0:0) | SLM:000063777 |
| Phosphatidylcholine (18:0_0:0) | SLM:000063933 |
| Phosphatidylcholine (18:1_0:0) | SLM:000063983 |
| Phosphatidylcholine (18:2_0:0) | SLM:000064032 |
| Phosphatidylcholine (20:3_0:0) | SLM:000064347 |
| Phosphatidylcholine (20:4_0:0) | SLM:000064388 |
| Phosphatidylcholine (22:6_0:0) | SLM:000064712 |
| Phosphatidylethanolamine (16:0_0:0) | SLM:000067687 |
| Phosphatidylethanolamine (18:0_0:0) | SLM:000067897 |
| Phosphatidylethanolamine (18:1_0:0) | SLM:000067947 |
| Phosphatidylethanolamine (18:2_0:0) | SLM:000067996 |
| Phosphatidylethanolamine (20:4_0:0) | SLM:000068352 |
| Phosphatidylcholine (14:0_16:0) | SLM:000063559 |
| Phosphatidylcholine (14:0_18:1) | SLM:000063564 |
| Phosphatidylcholine (14:0_18:2) | SLM:000063565 |
| Phosphatidylcholine (15:0_18:1) | SLM:000063675 |
| Phosphatidylcholine (15:0_18:2) | SLM:000063676 |
| Phosphatidylcholine (16:0_16:0) | SLM:000063724 |
| Phosphatidylcholine (16:0_16:1) | SLM:000063725 |
| Phosphatidylcholine (16:0_17:1) |  |
| Phosphatidylcholine (16:0_18:0) | SLM:000063728 |
| Phosphatidylcholine (16:0_18:1) | SLM:000063729 |
| Phosphatidylcholine (16:0_18:2) | SLM:000063730 |
| Phosphatidylcholine (16:0_18:3) | SLM:000063731 |
| Phosphatidylcholine (16:0_20:1) | SLM:000063735 |
| Phosphatidylcholine (16:0_20:2) | SLM:000063736 |
| Phosphatidylcholine (16:0_20:3) | SLM:000063737 |
| Phosphatidylcholine (16:0_20:4) | SLM:000063738 |
| Phosphatidylcholine (16:0_20:5) | SLM:000063739 |
| Phosphatidylcholine (16:0_22:4) | SLM:000063745 |
| Phosphatidylcholine (16:0_22:5) | SLM:000063746 |
| Phosphatidylcholine (16:0_22:6) | SLM:000063747 |
| Phosphatidylcholine (16:1_18:0) | SLM:000063781 |
| Phosphatidylcholine (16:1_18:1) | SLM:000063782 |
| Phosphatidylcholine (16:1_18:2) | SLM:000063783 |
| Phosphatidylcholine (16:1_20:4) | SLM:000063791 |
| Phosphatidylcholine (17:0_18:2) | SLM:000063886 |
| Phosphatidylcholine (17:0_20:3) | SLM:000063893 |
| Phosphatidylcholine (17:0_20:4) | SLM:000063894 |
| Phosphatidylcholine (18:0_18:1) | SLM:000063935 |
| Phosphatidylcholine (18:0_18:2) | SLM:000063936 |
| Phosphatidylcholine (18:0_18:3) | SLM:000063937 |
| Phosphatidylcholine (18:0_20:2) | SLM:000063942 |
| Phosphatidylcholine (18:0_20:3) | SLM:000063943 |
| Phosphatidylcholine (18:0_20:4) | SLM:000063944 |
| Phosphatidylcholine (18:0_20:5) | SLM:000063945 |
| Phosphatidylcholine (18:0_22:5) | SLM:000063952 |
| Phosphatidylcholine (18:0_22:6) | SLM:000063953 |
| Phosphatidylcholine (18:1_18:1) | SLM:000063984 |
| Phosphatidylcholine (18:1_18:2) | SLM:000063985 |
| Phosphatidylcholine (18:1_18:3) | SLM:000063986 |
| Phosphatidylcholine (18:1_20:2) | SLM:000063991 |
| Phosphatidylcholine (18:1_20:3) | SLM:000063992 |
| Phosphatidylcholine (18:1_20:4) | SLM:000063993 |
| Phosphatidylcholine (18:2_18:2) | SLM:000064033 |
| Phosphatidylcholine (18:2_20:3) | SLM:000064040 |
| Phosphatidylcholine (18:2_20:4) | SLM:000064041 |
| Phosphatidylcholine (O-16:0_16:0) | SLM:000065919 |
| Phosphatidylcholine (O-16:0_16:1) | SLM:000065920 |
| Phosphatidylcholine (O-16:0_18:1) | SLM:000065924 |
| Phosphatidylcholine (O-16:0_18:2) | SLM:000065925 |
| Phosphatidylcholine (O-16:0_20:3) | SLM:000065932 |
| Phosphatidylcholine (O-16:0_20:4) | SLM:000065933 |
| Phosphatidylcholine (O-16:1_16:0) | SLM:000065984 |
| Phosphatidylcholine (O-16:1_18:0) | SLM:000065988 |
| Phosphatidylcholine (O-16:1_18:1) | SLM:000065989 |
| Phosphatidylcholine (O-16:1_18:2) | SLM:000065990 |
| Phosphatidylcholine (O-16:1_20:3) | SLM:000065997 |
| Phosphatidylcholine (O-16:1_20:4) | SLM:000065998 |
| Phosphatidylcholine (O-16:2_18:0) |  |
| Phosphatidylcholine (O-17:0_15:0) | SLM:000066048 |
| Phosphatidylcholine (O-17:0_17:1) |  |
| Phosphatidylcholine (O-18:0_14:0) | SLM:000066176 |
| Phosphatidylcholine (O-18:0_16:1) | SLM:000066180 |
| Phosphatidylcholine (O-18:0_20:4) | SLM:000066193 |
| Phosphatidylcholine (O-18:1_16:0) | SLM:000066244 |
| Phosphatidylcholine (O-18:1_18:2) | SLM:000066250 |
| Phosphatidylcholine (O-18:1_20:3) | SLM:000066257 |
| Phosphatidylcholine (O-18:1_20:4) | SLM:000066258 |
| Phosphatidylcholine (O-18:2_16:0) | SLM:000066309 |
| Phosphatidylcholine (O-18:2_18:1) | SLM:000066314 |
| Phosphatidylcholine (O-18:2_18:2) | SLM:000066315 |
| Phosphatidylcholine (O-18:2_20:4) | SLM:000066323 |
| Phosphatidylethanolamine (16:0_18:2) | SLM:000067694 |
| Phosphatidylethanolamine (16:0_20:4) | SLM:000067702 |
| Phosphatidylethanolamine (18:0_18:2) | SLM:000067900 |
| Phosphatidylethanolamine (18:0_20:4) | SLM:000067908 |
| Phosphatidylethanolamine (18:1_18:1) | SLM:000067948 |
| Phosphatidylethanolamine (O-16:1_18:2) | SLM:000069954 |
| Phosphatidylethanolamine (O-16:1_20:4) | SLM:000069962 |
| Phosphatidylethanolamine (O-18:1_18:2) | SLM:000070214 |
| Phosphatidylethanolamine (O-18:1_20:4) | SLM:000070222 |
| Phosphatidylethanolamine (O-18:2_18:1) | SLM:000070278 |
| Phosphatidylethanolamine (O-18:2_18:2) | SLM:000070279 |
| Phosphatidylethanolamine (O-18:2_20:4) | SLM:000070287 |
| Phosphatidylinositol (16:0_18:1) | SLM:000073801 |
| Phosphatidylinositol (16:0_18:2) | SLM:000073802 |
| Phosphatidylinositol (16:0_20:4) | SLM:000073810 |
| Phosphatidylinositol (18:0_18:1) | SLM:000074007 |
| Phosphatidylinositol (18:0_18:2) | SLM:000074008 |
| Phosphatidylinositol (18:0_20:3) | SLM:000074015 |
| Phosphatidylinositol (18:0_20:4) | SLM:000074016 |
| Phosphatidylinositol (18:1_18:1) | SLM:000074056 |
| Phosphatidylinositol (18:1_18:2) | SLM:000074057 |
| Phosphatidylinositol (18:1_20:4) | SLM:000074065 |
| Phosphatidylinositol (18:2_18:2) | SLM:000074105 |
| Sphingomyelin (d32:1) | SLM:000390695 |
| Sphingomyelin (d34:0) | SLM:000390716 |
| Sphingomyelin (d34:1) | SLM:000390714 |
| Sphingomyelin (d34:2) | SLM:000390712 |
| Sphingomyelin (d36:1) | SLM:000390739 |
| Sphingomyelin (d36:2) | SLM:000390737 |
| Sphingomyelin (d38:1) | SLM:000390767 |
| Sphingomyelin (d38:2) | SLM:000390765 |
| Sphingomyelin (d40:1) | SLM:000390797 |
| Sphingomyelin (d40:2) | SLM:000390795 |
| Sphingomyelin (d42:2) | SLM:000390823 |
| Triacylglycerol (46:1) | SLM:000308244 |
| Triacylglycerol (46:2) | SLM:000308245 |
| Triacylglycerol (48:0) | SLM:000308257 |
| Triacylglycerol (48:1) | SLM:000308258 |
| Triacylglycerol (48:2) | SLM:000308259 |
| Triacylglycerol (48:3) | SLM:000308260 |
| Triacylglycerol (49:1) | SLM:000308267 |
| Triacylglycerol (49:2) | SLM:000308268 |
| Triacylglycerol (50:1) | SLM:000308276 |
| Triacylglycerol (50:2) | SLM:000308277 |
| Triacylglycerol (50:3) | SLM:000308278 |
| Triacylglycerol (50:4) | SLM:000308279 |
| Triacylglycerol (50:5) | SLM:000308280 |
| Triacylglycerol (51:1) | SLM:000308286 |
| Triacylglycerol (51:2) | SLM:000308287 |
| Triacylglycerol (51:3) | SLM:000308288 |
| Triacylglycerol (51:4) | SLM:000308289 |
| Triacylglycerol (52:2) | SLM:000308298 |
| Triacylglycerol (52:3) | SLM:000308299 |
| Triacylglycerol (52:4) | SLM:000308300 |
| Triacylglycerol (52:5) | SLM:000308301 |
| Triacylglycerol (52:6) | SLM:000308302 |
| Triacylglycerol (53:2) | SLM:000308309 |
| Triacylglycerol (53:3) | SLM:000308310 |
| Triacylglycerol (53:4) | SLM:000308311 |
| Triacylglycerol (54:3) | SLM:000308323 |
| Triacylglycerol (54:4) | SLM:000308324 |
| Triacylglycerol (54:5) | SLM:000308325 |
| Triacylglycerol (54:6) | SLM:000308326 |
| Triacylglycerol (54:7) | SLM:000308327 |
| Triacylglycerol (56:3) | SLM:000308349 |
| Triacylglycerol (56:4) | SLM:000308350 |
| Triacylglycerol (56:5) | SLM:000308351 |
| Triacylglycerol (56:6) | SLM:000308352 |
| Triacylglycerol (56:7) | SLM:000308353 |
| Triacylglycerol (56:8) | SLM:000308354 |
| Triacylglycerol (58:7) | SLM:000308381 |
| Triacylglycerol (58:8) | SLM:000308382 |
| Triacylglycerol (58:9) | SLM:000308383 |

**Table S2.** Proportion of explained variance for the first 5 PCs in each biomarker layer (Metabolite, Lipid and Protein). The proportion of explained variance is presented for the principal component analysis performed for the obese participants (n= 416) (MHO+MUO) and the non-MUO (MUO+NOC) (n= 3,027).

|  | **Obesity (MHO + MUO)** | | |  | **Non-MUO (MHO + NOC)** | |  |
| --- | --- | --- | --- | --- | --- | --- | --- |
| **Principal component** | **Metabolite PCA** | **Lipid PCA** | **Protein PCA** | | **Metabolite PCA** | **Lipid PCA** | **Protein PCA** |
| **PC1** | 0,224421641 | 0,358244711 | 0,310839942 | | 0,230624733 | 0,310291 | 0,309865 |
| **PC2** | 0,301968337 | 0,491094184 | 0,406591967 | | 0,31999479 | 0,45742 | 0,406745 |
| **PC3** | 0,344817802 | 0,548965328 | 0,470603332 | | 0,358183646 | 0,519477 | 0,47723 |
| **PC4** | 0,382685351 | 0,601458597 | 0,502824769 | | 0,393781876 | 0,572801 | 0,509675 |
| **PC5** | 0,418152925 | 0,638306047 | 0,530748343 | | 0,429899156 | 0,614066 | 0,534675 |

**Table S3.** Loadings of metabolite PCs 1-5 (MHO vs MUO).

| **Metabolite** | **PC1** | **PC2** | **PC3** | **PC4** | **PC5** |
| --- | --- | --- | --- | --- | --- |
| **X1.Methyladenosine** | 0.118342789854631 | -0.0429478220911972 | -0.156183393155615 | -0.0673688858409903 | 0.0347624041002515 |
| **X1.Methylhistidine** | 0.131580338368253 | 0.0545889453986746 | -0.212851853862449 | 0.0145862605416955 | -0.0194160511989565 |
| **X25.hydroxyvitamin.D3** | 0.022374214957299 | 0.0102391510614677 | 0.0528281214640669 | 0.0918282491849864 | 0.0565073512469733 |
| **X2.Aminoisobutyrate** | 0.068713720914363 | 0.0440560021649749 | 0.137139221298411 | 0.126544860725479 | -0.00725994109603775 |
| **X3.Hydroxytrimethyllysine** | 0.118081857577641 | 0.0713033324389121 | -0.160476618263753 | -0.0389692117367248 | -0.0143226437851216 |
| **X3.Methylhistidine** | 0.00963143224344849 | 0.0216389888633892 | -0.063649376959081 | 0.059721487872937 | -0.0682732054496329 |
| **X4.Trimethylammoniobutanoate** | 0.132688592318026 | 0.0531624308566197 | -0.0602962381975206 | -0.102010128064806 | -0.1919076855534 |
| **X5.Methylthioadenosine** | 0.100977346140002 | 0.0108839970192683 | 0.027886973657975 | -0.338751338489967 | 0.0558645882855145 |
| **X7.Methylguanine** | 0.134420406293939 | 0.0462721854603149 | -0.0903007554593383 | -0.146811993383442 | -0.112700866171894 |
| **AAMU** | 0.0615826211835808 | 0.0120673517251129 | -0.144969657542094 | -0.13677675653674 | 0.0396274953823597 |
| **Acetylarginine** | 0.082227903085813 | 0.0714610043597805 | 0.0291935918885623 | 0.0378359113898502 | 0.127464852409102 |
| **Acetylcarnitine** | 0.126819833595638 | -0.110159431889981 | 0.00854639418374202 | 0.035947247516489 | 0.108295453372387 |
| **Acetylornithine** | 0.0571913437942091 | 0.00798080770827457 | -0.17726494701707 | -0.0123115462312191 | -0.0241110770290615 |
| **Acisoga** | 0.09356368312537 | -0.0360617843071313 | -0.138335321908545 | 0.0696362577334133 | 0.0517986010527628 |
| **ADMA** | 0.0852798875866472 | -0.00687341193260936 | -0.0663318763131331 | -0.0571769170783989 | 0.117316605010694 |
| **Alanine** | 0.084102927233492 | 0.1313772521578 | 0.0974331121896249 | 0.0560457076454581 | 0.0783833923091118 |
| **Arginine** | 0.0965864985991276 | 0.0731956264929308 | 0.00167644261069069 | -0.0512938372627705 | 0.102410454619257 |
| **Asparagine** | 0.122315686330528 | 0.0953686453249496 | 0.0941862235549434 | -0.134744824163799 | 0.142959378301745 |
| **Beta.carotene** | 0.0325764057525971 | 0.0264423372146177 | -0.068597441452742 | -0.0834799828244821 | 0.0188981454924901 |
| **Betaine** | 0.115313496843616 | 0.0274404660668792 | 0.0315667480108304 | -0.0119202879071593 | -0.00982361891556274 |
| **Butyrylcarnitine** | 0.0778613127631958 | 0.0413619608656616 | -0.0703713225195088 | 0.10904450897978 | 0.0172186600134622 |
| **C10.0.carnitine** | 0.0912514689284518 | -0.154561609601032 | 0.052282910168449 | 0.013891460178534 | -0.0552284588694025 |
| **C10.0.OH.carnitine** | 0.122755758751142 | -0.152818319123081 | 0.0333138385758359 | 0.0386030136891557 | -0.127308330687117 |
| **C10.1.carnitine** | 0.114104215403848 | -0.161390420153798 | 0.0283018469853504 | 0.0248613714379241 | -0.0283675255144424 |
| **C10.2.carnitine** | 0.129601872349272 | -0.132416896390114 | -0.0504501537985632 | 0.0500411559731633 | -0.0508012762652095 |
| **C10.3.Carnitine** | 0.0995409277534533 | -0.105160811025697 | -0.0693295298790396 | 0.0855401826229455 | 0.0130873384994335 |
| **C11.0.carnitine** | 0.126310225104173 | -0.108127411249617 | 0.0264144008517132 | 0.0120045432637261 | -0.0903796781619041 |
| **C11.1.carnitine** | 0.14646760597986 | -0.118325566730482 | -0.0212219380093712 | 0.0958160545941006 | -0.0506997835504167 |
| **C12.0.carnitine** | 0.120915207487741 | -0.183458432186418 | 0.0857772249807026 | 0.0155731758405268 | -0.0465621952429596 |
| **C12.1.carnitine** | 0.121118170019666 | -0.206930063882398 | 0.059077992436127 | 0.0282815026233048 | -0.0136440855972516 |
| **C12.2.carnitine** | 0.13090511648351 | -0.172844592068429 | 0.0290541836550535 | 0.0359560958868504 | -0.0476439553052122 |
| **C13.0.carnitine** | 0.0904826963759627 | -0.0960374795867637 | 0.0208290933520939 | 0.00384844471639035 | -0.0545510627268438 |
| **C13.1.carnitine** | 0.123127004979364 | -0.116567100521381 | 0.0148566093148115 | 0.0409975162425444 | -0.0716083547831656 |
| **C14.1.carnitine** | 0.11015952753307 | -0.203775765125046 | 0.0772923269664015 | 0.017947436443926 | 0.0076963576528703 |
| **C14.2.carnitine** | 0.105435583673952 | -0.18341211437177 | 0.0581181789986581 | 0.0201211821413691 | -0.0122285275419335 |
| **C16.1.carnitine** | 0.110383300287283 | -0.205395538457142 | 0.0818590259932354 | 0.00886934561788553 | 0.105783090931322 |
| **C18.0.carnitine** | 0.107273160309839 | -0.0758157136648714 | -0.000653999989533951 | -0.0372389072730983 | 0.0805735517870651 |
| **C18.1.carnitine** | 0.132445146766239 | -0.153012796504715 | 0.045896579905138 | -0.0290676929407087 | 0.111955438754969 |
| **C18.2.carnitine** | 0.127989477916901 | -0.144650736648232 | 0.0535757477823048 | -0.0207940240368779 | 0.108912011582312 |
| **C4.0.OH.carnitine** | -0.00515975114791564 | -0.0339585241049208 | 0.0447871226561836 | 0.0226770943802861 | 0.011364258297037 |
| **C6.0.carnitine** | 0.0933040434387452 | -0.149833529454558 | 0.0416339081336472 | 0.0347997073023341 | -0.00880871952575072 |
| **C8.0.OH.carnitine** | 0.108053799364721 | -0.14261096445363 | 0.0476938482853042 | -0.014575670624639 | -0.121475633108727 |
| **C8.1.carnitine** | 0.117964727325797 | -0.119510371655918 | -0.0302893664227793 | 0.102531248620303 | 0.0404014890693125 |
| **C9.0.carnitine** | 0.0875925382285677 | -0.0530189878339042 | -0.0432219420856847 | 0.00326230424788569 | -0.0889346647429916 |
| **Caffeine** | 0.0513581461755396 | 0.0406041905236573 | -0.000200211026086738 | 0.00223836203117229 | -0.025755049812434 |
| **Carnitine** | 0.0651369568555005 | 0.0200929553942311 | -0.0142950889159166 | 0.0343856849181762 | 0.0762608817751386 |
| **Choline** | 0.0773153797497619 | 0.0354451191296335 | -0.0112147890694673 | 0.194729789774399 | 0.10273792822937 |
| **Citrulline** | 0.1229928967172 | 0.0687817403414523 | -0.0855191006837156 | -0.135174289467176 | 0.163586791343286 |
| **Cotinine** | 0.0334375727649869 | -0.0465556752787667 | 0.00713713253007677 | -0.116362367668482 | -0.108066432991617 |
| **Creatine** | 0.0140740560501274 | 0.0274370841319354 | -0.0373407767644432 | 0.140075978521413 | 0.287177928825025 |
| **Creatinine** | 0.146171570980038 | 0.0727816516876778 | -0.0796237334598656 | -0.121892697479197 | -0.240535291323137 |
| **Cystine** | 0.131838588441408 | 0.0341254048138205 | -0.0253042962665125 | -0.179648658203101 | 0.0344507535745419 |
| **Dimethylglycine** | 0.0602600814643571 | -0.00623652855220674 | -0.0643834560110275 | 0.0468454293248676 | -0.0476529863288012 |
| **Dimethyllysine** | 0.0581601018560361 | 0.0164881263839223 | -0.223930748922815 | -0.0127683183264833 | -0.0234141884787206 |
| **DMGV** | 0.0680370403748061 | 0.153341794068776 | -0.127350416494221 | 0.0365805734926543 | -0.0703946605953178 |
| **Ergothioneine** | 0.0306484856362955 | 0.0496184338591797 | -0.027093811330404 | 0.0648542706205797 | -0.101270531063029 |
| **Glutamate** | -0.0418434546284199 | -0.0149139801946613 | -0.00323063013193732 | 0.295432300474216 | 0.0693901436980601 |
| **Glutamine** | 0.121161238384607 | 0.0630040126410235 | 0.0463141785717525 | -0.305962208174506 | 0.0431226889300834 |
| **Glycerophosphocholine** | -0.00595942229740489 | 0.0574896016009807 | 0.0721764982731776 | 0.0834374860776264 | 0.105991833807083 |
| **Guanidineacetate** | 0.0678337537833452 | 0.0438691786219142 | 0.10353423459232 | -0.172114126745447 | -0.135981841785651 |
| **Hippurate** | 0.0469595447358892 | 0.0565364807039987 | -0.154733007008477 | -0.0122402438323027 | 0.0447646670731622 |
| **Histidine** | 0.123607685853523 | 0.107564317988609 | 0.117628638204596 | -0.0546582973428523 | 0.13538922707692 |
| **Homoarginine** | 0.0648116986224491 | 0.115915153013146 | 0.210844248408558 | 0.0767255974672728 | -0.0974857210128016 |
| **Homocitrulline** | 0.0428709411675434 | 0.0343103999356075 | -0.217362661558175 | 0.126234846571809 | 0.0884961097711439 |
| **Homostachydrine** | 0.0443286200327407 | -0.0188726332564255 | -0.0564574612075312 | 0.00634454623355304 | 0.0663635090479026 |
| **Hydroxycotinine** | 0.0574109272634162 | 0.00491596722942809 | -0.0836052477638773 | 0.0679050974374205 | 0.149383062452798 |
| **Hypoxanthine** | 0.0424032496056072 | 0.0237908702038833 | -0.0675132245765106 | 0.172112502440015 | 0.112225606001687 |
| **Isoleucine** | 0.119167471910717 | 0.216085279168725 | 0.137189154228228 | 0.110178416298782 | -0.132632750531696 |
| **Isovalerylcarnitine** | 0.124680772314024 | 0.0891010940512617 | -0.0267555746522622 | 0.158677111841643 | -0.0622744055077232 |
| **Kynurenate** | 0.115298131214354 | 0.0845814747300909 | -0.0573896744885241 | 0.076043789989563 | -0.120026487838706 |
| **Kynurenine** | 0.137498302418558 | 0.0738271743088597 | -0.105430548658213 | 0.0152464081685158 | -0.0419663081493325 |
| **Leucine** | 0.126284956240221 | 0.201525504311819 | 0.1734396248653 | 0.123992096829326 | -0.126346718719889 |
| **Lysine** | 0.122819049444148 | 0.10775971840833 | 0.14999996098669 | 0.0371181190020236 | 0.0927065495264199 |
| **Methionine** | 0.137735440363862 | 0.120468430540994 | 0.1362491925447 | -0.0408521296670502 | 0.0264402520608142 |
| **Methionine.S.oxide** | 0.0334056096559772 | 0.0466627756153527 | 0.0148046118622827 | 0.0682375105237986 | 0.0460961902057851 |
| **Methyllysine** | 0.0286512491752618 | -0.00443793609451005 | -0.188533979420615 | -0.028164208757396 | -0.00447693109189182 |
| **Methylnicotinamide** | 0.0483218239091242 | 0.0423192016009993 | 0.0321051520268075 | 0.0538613386847627 | -0.00780123945380176 |
| **Myristoylcarnitine** | 0.136098381844778 | -0.145121534749171 | 0.0818366728728734 | 0.010626967364147 | 0.0220142981620154 |
| **N2.N2.Dimethylguanosine** | 0.126066810768169 | 0.0631163038402147 | -0.112258583743626 | -0.126894233574803 | -0.0999985816059727 |
| **N.Acetylcarnosine** | 0.0971689932472328 | 0.0724730373543541 | 0.0507375990938954 | -0.0329523637919505 | -0.252131992821513 |
| **Nicotinamide** | 0.0563838851790059 | 0.0617460933047538 | 0.0353516886881185 | 0.137327182303883 | 0.054360430637875 |
| **N.Methyl.2.pyridone.5.carboxamide** | 0.0544225719398576 | 0.0183029365031972 | -0.0889122874068323 | 0.138506714221896 | -0.0166418351072456 |
| **N.Methyl.4.pyridone.3.carboxamide** | 0.0614916737199625 | 0.0293746240837273 | -0.0910173426599776 | 0.111961457250232 | -0.018590424733509 |
| **N.Methylproline** | 0.01032133629548 | 0.0357622347534881 | -0.112388139531047 | -0.00143043502062111 | 0.00422886929589143 |
| **NMMA** | 0.0701058047584638 | 0.0548489093142981 | 0.0642753681404149 | -0.0349308401700845 | 0.197460222509529 |
| **Octanoylcarnitine** | 0.0853998773855757 | -0.14144310165551 | 0.0527643265157416 | 0.0182250214831613 | -0.0413498818906533 |
| **Ornithine** | 0.1121067904949 | 0.0800504359066116 | 0.0586285540952126 | -0.0478231437642361 | 0.13916498634854 |
| **Palmitoylcarnitine** | 0.148816525306544 | -0.0874046092094796 | 0.0923550298858639 | 0.0171401386837274 | 0.0507533472387761 |
| **Pantothenate** | 0.0449535745827002 | 0.022270565958122 | -0.00948098188203308 | 0.141057025799818 | 0.053191860369152 |
| **Paraxanthine** | 0.0799953111689663 | 0.0308441639651575 | -0.0338639518469049 | -0.0579778437219313 | -0.00543998750281008 |
| **Phenylacetylglutamine** | 0.071430108831038 | 0.0399638058566232 | -0.15964681896667 | -0.0356047743563319 | 0.110243026151163 |
| **Phenylalanine** | 0.158322909282139 | 0.144423720850977 | 0.114837223204621 | 0.0299339518320095 | -0.0267113889400907 |
| **Pipecolate** | 0.0111306961490209 | 0.0645568325959606 | -0.011390256928046 | 0.0366263777915966 | -0.0219733352957702 |
| **Piperine** | 0.0235786237276346 | 0.0390295067083838 | -0.00736849395650635 | 0.0516770517698181 | -0.109636913346651 |
| **Proline** | 0.0974997870050435 | 0.157249291725408 | 0.0249989571039865 | -0.0348036764741324 | 0.029012237833499 |
| **Proline.betaine** | 0.0137392759426967 | 0.0207764245342856 | -0.137747695317186 | 0.00317257999367274 | 0.0345803294721202 |
| **Propionylcarnitine** | 0.119714316609911 | 0.080204390367367 | -0.0939421506339888 | 0.154149708471657 | 0.0259865864740315 |
| **Pyroglutamate** | 0.0740067247606206 | 0.0314903334504071 | 0.0903964897032757 | -0.0613573095966676 | 0.0782678385103488 |
| **Sarcosine** | -0.00172982080881638 | 0.0306949740015523 | -0.0477654568226054 | 0.100590844467001 | 0.19127213272749 |
| **SDMA** | 0.113608457745072 | 0.0285756818020995 | -0.0644869547353146 | -0.0656068807930128 | 0.148154932679941 |
| **Serine** | 0.0738846546810947 | 0.00410176984355226 | 0.0477105426045317 | -0.13155705164678 | 0.242308688767714 |
| **Taurine** | 0.10407705392418 | 0.0146619227161219 | -0.10483178296936 | 0.0362054809119084 | 0.145878884531918 |
| **Threonine** | 0.0667152773954594 | 0.0997978623192607 | 0.129811685876452 | -0.0897154669432217 | 0.0872837979982605 |
| **Tiglylcarnitine** | 0.106153450505892 | 0.0219326076573176 | -0.145647671907584 | 0.101764392981114 | -0.0776500168508356 |
| **Trigonelline** | 0.00923666362788535 | -0.0130683044325014 | -0.172965052923437 | -0.0466375145719918 | 0.0442264314987213 |
| **Trimethylamine.N.oxide** | 0.020863488721665 | 0.037804144480221 | -0.0542708205754712 | 0.074559500870937 | -0.0198219535619016 |
| **Trimethyllysine** | 0.0779459425236348 | 0.0579426084757522 | -0.0810140559566582 | 0.000644680710204197 | -0.0756934075915862 |
| **Tryptophan** | 0.124340459145199 | 0.162332136081173 | 0.166512857831647 | 0.0704001616845222 | 0.00282179505993872 |
| **Tyrosine** | 0.0748733526029275 | 0.0761046527180216 | 0.0840642136537702 | 0.0256085193153418 | -0.0592279313896742 |
| **Urea** | 0.102146114377241 | 0.0641156906308028 | -0.141041593197507 | 0.0704824529430874 | -0.0493825837119572 |
| **Urocanate** | 0.0189648787094257 | 0.00523590298988158 | -0.0349845182920457 | 0.0261359127627805 | 0.0216325916122248 |
| **Valine** | 0.105878339404438 | 0.180815389863071 | 0.113874986817912 | 0.109246889618321 | -0.0514372091674871 |

**Table S4.** Loadings of lipid PCs 1-5 (MHO vs MUO)

| **Lipid** | **PC1** | **PC2** | **PC3** | **PC4** | **PC5** |
| --- | --- | --- | --- | --- | --- |
| **CE 14:0;0** | 0.0971798363986408 | 0.011276149053025 | 0.130602187374526 | 0.0892867927404617 | -0.0393761240120183 |
| **CE 15:0;0** | 0.0486106174169783 | 0.0377132997826531 | 0.0731343675524837 | 0.024885239930484 | -0.0187482151119502 |
| **CE 16:0;0** | 0.101518146708807 | 0.0639874450286243 | 0.0947674487055743 | -0.0777963774242018 | 0.010722455521483 |
| **CE 16:1;0** | 0.0802945632858635 | 0.00433550085602845 | 0.151125439502099 | 0.0554513763822727 | -0.0227047940159382 |
| **CE 17:0;0** | 0.0601510261034761 | 0.0641475004181861 | 0.0493539919301312 | -0.02572693226714 | -0.00753971416719309 |
| **CE 17:1;0** | 0.0836777732202895 | 0.0617838333285481 | 0.136650577013688 | 0.0148997338042148 | -0.033011494270328 |
| **CE 18:0;0** | 0.0783478892101671 | 0.0428490500870536 | -0.0332127848226689 | -0.0690104897871399 | -0.0275565606441928 |
| **CE 18:1;0** | 0.0972777228307806 | 0.0693973899626709 | 0.0958672346279421 | -0.048167795464903 | -0.0361897384120356 |
| **CE 18:2;0** | 0.0829046628829696 | 0.10305020953685 | -0.0347447731830829 | -0.0546735058636234 | -0.0345434370502449 |
| **CE 18:3;0** | 0.104098177264213 | 0.0240604403231515 | 0.0944965909837871 | 0.0424219766617035 | -0.0140520677267829 |
| **CE 20:2;0** | 0.0485820420155421 | 0.0531148938779424 | -0.0379594816880651 | -0.00534889992218223 | 0.00568710004954927 |
| **CE 20:3;0** | 0.0932697909915886 | 0.0326612365124254 | 0.037607314960386 | -0.0105420495164953 | -0.0086495952770177 |
| **CE 20:4;0** | 0.06559547440838 | 0.0657175427353209 | 0.0625748793286706 | -0.157429119195388 | 0.143442244646104 |
| **CE 20:5;0** | 0.0322571431957255 | 0.0252136729420057 | 0.2276401681101 | -0.0835636652643568 | 0.0248396220382687 |
| **CE 22:6;0** | 0.0468629597233617 | 0.0529216374490933 | 0.150934064259375 | -0.137073687977848 | 0.0294577095860121 |
| **Cer 40:1;2** | 0.0933548422560868 | 0.0190540184625179 | -0.0414815492615022 | 0.00405206186816079 | -0.0940987279403412 |
| **Cer 40:2;2** | 0.0783949128854477 | 0.0333509614131287 | -0.0202525384980622 | 0.00334060742888946 | -0.0599964740554514 |
| **Cer 42:1;2** | 0.0845155352782877 | 0.0319110321413479 | -0.0227507826409049 | -0.0111431804359306 | -0.0658022322654139 |
| **Cer 42:2;2** | 0.0958414234039078 | 0.03423207287441 | -0.00170431615709319 | -0.0836377681101541 | -0.0482482708333888 |
| **Chol** | 0.0383672762693309 | 0.0272247090131849 | 0.0126168769830636 | -0.0272444043537069 | -0.0350982829347175 |
| **DAG 16:0;0_18:1;0** | 0.0881203558436965 | -0.105160162149485 | -0.0178541090271333 | 0.0389666345076273 | 0.0390868857185501 |
| **DAG 16:0;0_18:2;0** | 0.0845879679418767 | -0.111132443150776 | -0.0768953785389832 | 0.0096991852040466 | 0.0511201855549889 |
| **DAG 16:1;0_18:1;0** | 0.0856306922603499 | -0.0842263522720105 | 0.0104739512353157 | 0.0177090244393419 | 0.0231268371667085 |
| **DAG 18:1;0_18:1;0** | 0.0869244116034031 | -0.0863523082991087 | -0.0570572063993728 | -0.0554592742377158 | 0.0143693943099284 |
| **DAG 18:1;0_18:2;0** | 0.0971531725810004 | -0.0958894819408099 | -0.117752280466687 | -0.0830596203920606 | 0.0226839146760479 |
| **DAG 18:1;0_18:3;0** | 0.076043722019424 | -0.0317800494131862 | -0.0280534985143768 | -0.0248083931309498 | 0.0174772547254894 |
| **LPC 14:0;0** | 0.053342092325645 | -0.00576962360576862 | 0.0932096548912491 | 0.198920621802379 | 0.145168721062949 |
| **LPC 16:0;0** | 0.0431793697651006 | 0.00230237410772578 | 0.00947251003106712 | 0.166616735537938 | 0.27737055995246 |
| **LPC 16:1;0** | 0.0465404585557408 | 0.00935768825264048 | 0.0380743720618306 | 0.162055891264821 | 0.207459690567394 |
| **LPC 18:0;0** | 0.035876859146098 | 0.0347458911448497 | -0.0472874436702058 | 0.120297270880815 | 0.236573237373443 |
| **LPC 18:1;0** | 0.0294770571049115 | 0.0562145351020708 | -0.0366448868050511 | 0.129575813543456 | 0.181834275618501 |
| **LPC 18:2;0** | 0.0203802010076378 | 0.0606437067153067 | -0.0744527807554908 | 0.0859377657649876 | 0.0900784701498788 |
| **LPC 20:3;0** | 0.0545811259561831 | 0.015157862279715 | -0.00768606433780354 | 0.142010935597091 | 0.186646230717199 |
| **LPC 20:4;0** | 0.0237455971684582 | 0.0234260349378427 | 0.0098159409174908 | 0.0313732070059583 | 0.307662393128169 |
| **LPC 22:6;0** | 0.0225892162717206 | 0.0261355204283993 | 0.112034271929466 | -0.0133294878154173 | 0.170960272002491 |
| **LPE 16:0;0** | 0.0688573501579958 | -0.0042769674835058 | 0.0358211122816914 | 0.136729732453934 | 0.142418771274785 |
| **LPE 18:0;0** | 0.0797101242736713 | -0.0137777734492022 | -0.0177644288993921 | 0.144933044032967 | 0.219214212234016 |
| **LPE 18:1;0** | 0.0563785366181474 | 0.0148364464593301 | -0.0529876163639652 | 0.114026997863494 | 0.0518154622370856 |
| **LPE 18:2;0** | 0.0573631511005799 | 0.04153951115659 | -0.0853562614116096 | 0.139438484546488 | -0.0127614341097178 |
| **LPE 20:4;0** | 0.0611681698149316 | 0.0435847365071017 | -0.0391879047214716 | 0.0331876281876018 | 0.124239847767605 |
| **PC 14:0;0_16:0;0** | 0.0713529676201919 | 0.0235963312796386 | 0.106642442642157 | 0.10094654977267 | -0.0455112566213799 |
| **PC 14:0;0_18:1;0** | 0.059965094186989 | 0.010549529365879 | 0.0466571702465243 | 0.0969333303149302 | -0.0481831230672054 |
| **PC 14:0;0_18:2;0** | 0.0673758193028659 | 0.0251685140714641 | 0.0225573569047992 | 0.058949594172545 | -0.0807150135821547 |
| **PC 15:0;0_18:1;0** | 0.0835342545299977 | 0.0261275485925682 | 0.0936800712702719 | 0.0553941237886455 | -0.0653903366021124 |
| **PC 15:0;0_18:2;0** | 0.0886190805624756 | 0.0578479610191904 | -0.0327952976272835 | 0.0480715333003888 | -0.107294134591576 |
| **PC 16:0;0_16:0;0** | 0.0857815018599483 | 0.0753076462796631 | 0.100921591707263 | -0.00437843589309193 | -0.0207279978148699 |
| **PC 16:0;0_16:1;0** | 0.0662639961301616 | 0.00252463536881489 | 0.115321906795766 | 0.0652420586391803 | -0.0649221600055943 |
| **PC 16:0;0_17:1;0** | 0.0479060887411656 | 0.00542216463473708 | 0.0600002271398327 | -0.00346089314675416 | -0.0443017161661286 |
| **PC 16:0;0_18:0;0** | 0.093816722615416 | 0.0785059887649514 | -0.0288317372453382 | 0.0141188314821679 | -0.0735732240692282 |
| **PC 16:0;0_18:1;0** | 0.093149998718701 | 0.0294854026471884 | 0.0972665571102027 | 0.0439427691718173 | -0.0694410019044417 |
| **PC 16:0;0_18:2;0** | 0.0989926983547233 | 0.0725894447857957 | -0.0521499196112748 | 0.0278045779217902 | -0.105705889451119 |
| **PC 16:0;0_18:3;0** | 0.0891787906753889 | 0.0226592400638539 | 0.0959115707492087 | 0.0877430790084439 | -0.0766019638331722 |
| **PC 16:0;0_20:1;0** | 0.0622250228257826 | 0.0233213256422775 | 0.142499665968045 | 0.0117795840472577 | -0.0484016187292112 |
| **PC 16:0;0_20:2;0** | 0.110723298348153 | 0.049808237378004 | 0.0189840048873729 | 0.0362704802418767 | -0.041550014268072 |
| **PC 16:0;0_20:3;0** | 0.10629945076357 | -0.00465585599628674 | 0.0168731330945512 | 0.0454210105480529 | -0.0221204829714494 |
| **PC 16:0;0_20:4;0** | 0.0773918719593799 | 0.024837777216773 | 0.0674722098463443 | -0.0756017727132815 | 0.111395656562673 |
| **PC 16:0;0_20:5;0** | 0.040274213548951 | 0.00983532240839321 | 0.208183399773236 | -0.0375153939370007 | 0.0199690215885243 |
| **PC 16:0;0_22:4;0** | 0.0861869971389769 | 0.0248479684262508 | 0.021334412276868 | 0.0254038371028901 | 0.0630536121616371 |
| **PC 16:0;0_22:5;0** | 0.0856028873767683 | 0.0133871984711054 | 0.163480119068864 | -0.0127255665097951 | 0.0446270370357202 |
| **PC 16:0;0_22:6;0** | 0.0590490939986641 | 0.0296028437996608 | 0.160897162310005 | -0.103630673284267 | 0.0348417734546151 |
| **PC 16:1;0_18:0;0** | 0.0693158786925205 | 0.000720845768461855 | 0.103944408597456 | 0.0900751163295889 | -0.0568748763599537 |
| **PC 16:1;0_18:1;0** | 0.0741696747039966 | 0.0549507660443292 | 0.0256941376097691 | 0.0470601916091579 | -0.0595233246909502 |
| **PC 16:1;0_18:2;0** | 0.0772989053751582 | 0.0689368105778016 | -0.0945345811209295 | 0.0576319733482096 | -0.0673931045200535 |
| **PC 16:1;0_20:4;0** | 0.0516799101720027 | 0.0579541522206927 | 0.0311881788638316 | -0.0450108076932297 | 0.100308052715734 |
| **PC 17:0;0_18:2;0** | 0.0783405221467223 | 0.0635812495390255 | -0.0663163961731549 | 0.0107533484722058 | -0.0836357542303073 |
| **PC 17:0;0_20:3;0** | 0.0819963209218811 | 0.0245785097007284 | -0.032936124737279 | 0.0045872348246305 | -0.03344201122216 |
| **PC 17:0;0_20:4;0** | 0.0858413699564797 | 0.0513044331363042 | 0.0178229633305963 | -0.0694039999243193 | 0.107837651363449 |
| **PC 18:0;0_18:1;0** | 0.0920362820125758 | 0.0430010344143682 | 0.0720058680288559 | 0.0647596797022163 | -0.0770023231991859 |
| **PC 18:0;0_18:2;0** | 0.0997670534353883 | 0.0868741324058898 | -0.0878687026039157 | 0.00427435168388041 | -0.0850706871599461 |
| **PC 18:0;0_18:3;0** | 0.0757712240498129 | 0.0316215263954763 | 0.109813071628157 | 0.0464645247652124 | -0.0435537105828794 |
| **PC 18:0;0_20:2;0** | 0.0809484369601726 | 0.0461114696286139 | 0.00100955851611803 | 0.00969076832262135 | -0.0767780617236468 |
| **PC 18:0;0_20:3;0** | 0.11032514141773 | -0.00524481622109435 | 0.00548191716734163 | 0.0296424390099349 | -0.0292883303566798 |
| **PC 18:0;0_20:4;0** | 0.0819816424570495 | 0.048866030774107 | 0.0296719628479296 | -0.125612798318019 | 0.121105124228999 |
| **PC 18:0;0_20:5;0** | 0.0454879317232644 | 0.0125908119619726 | 0.188763986485989 | -0.0434445939427784 | 0.0189238407643861 |
| **PC 18:0;0_22:5;0** | 0.0996926268197613 | -0.00344709531453675 | 0.105096187278701 | 0.0228534226797001 | 0.0110449427401867 |
| **PC 18:0;0_22:6;0** | 0.0726592223943086 | 0.0440890409724385 | 0.165950877220281 | -0.0931301217677159 | 0.0136707381677487 |
| **PC 18:1;0_18:1;0** | 0.0696258284809026 | 0.0770417769683764 | -0.0490071701520671 | 0.0337230993435729 | -0.0681121139198609 |
| **PC 18:1;0_18:2;0** | 0.0711229063737643 | 0.080065547407016 | -0.108881563965985 | 0.0418507202258646 | -0.0754647681216423 |
| **PC 18:1;0_18:3;0** | 0.0514707192295356 | 0.0307425272804722 | 0.0375140514373643 | 0.0374635024016082 | 0.00926089277082393 |
| **PC 18:1;0_20:2;0** | 0.0670437855917163 | 0.0400472717901648 | -0.0397086943147041 | 0.0593846525768368 | -0.0376507048956817 |
| **PC 18:1;0_20:3;0** | 0.0970431017258339 | 0.0347576475083978 | -0.0511548077054352 | 0.0138529395795041 | 0.0181140452655612 |
| **PC 18:1;0_20:4;0** | 0.078287815328617 | 0.0580271670380395 | 0.00799192875010766 | -0.0488293451269216 | 0.109156242365407 |
| **PC 18:2;0_18:2;0** | 0.0623766118387071 | 0.0763687421643756 | -0.11879632026534 | 0.0737388653338988 | -0.0440929302393557 |
| **PC 18:2;0_20:3;0** | 0.0757640683032902 | 0.0395733133874831 | -0.0608929128996319 | 0.0807613415815034 | -0.017968478120372 |
| **PC 18:2;0_20:4;0** | 0.0753186950248669 | 0.0794222565368828 | -0.047826838815807 | 0.01028644077523 | 0.0646110204678553 |
| **PC O-16:0;0/16:0;0** | 0.0331901549159261 | 0.0418534430609115 | -0.0102212525831802 | 0.109198883142583 | 0.158456597397199 |
| **PC O-16:0;0/16:1;0** | 0.0353430762161393 | 0.0301128732921633 | 0.0282739307752162 | 0.0423414109029125 | -0.00283499126411636 |
| **PC O-16:0;0/18:1;0** | 0.0490461896834929 | 0.115351132945217 | -0.0434683018374142 | 0.00431180923646739 | 0.00314348198138253 |
| **PC O-16:0;0/18:2;0** | 0.0361016866382856 | 0.133228459115639 | -0.107146355775685 | -0.0107761295218366 | 0.0267775113479704 |
| **PC O-16:0;0/20:3;0** | 0.0622683186442734 | 0.0895849838361032 | -0.0581162276906653 | -0.00748407114524603 | 0.0297558534530081 |
| **PC O-16:0;0/20:4;0** | 0.0441431434589821 | 0.0845653593099017 | -0.0367594848171962 | -0.117422092941237 | 0.119787059540082 |
| **PC O-16:1;0/16:0;0** | 0.0271513052064788 | 0.0769456757462447 | -0.00398360133057238 | 0.0581018946823536 | 0.167461646485815 |
| **PC O-16:1;0/18:0;0** | -0.012056936564738 | 0.0489743343873567 | -0.0820780572170276 | 0.0375893597775779 | 0.091036754369764 |
| **PC O-16:1;0/18:1;0** | 0.0141547164582807 | 0.122446759547143 | -0.0280503105938246 | -0.0119136046257085 | 0.0564847119194232 |
| **PC O-16:1;0/18:2;0** | 0.0397315230522427 | 0.125454057525228 | -0.048722418374279 | -0.0145181745650748 | 0.0194635490709003 |
| **PC O-16:1;0/20:3;0** | 0.0365101134358281 | 0.0531572920576593 | -0.0371620283589834 | -0.0405057018441937 | 0.0125695361736058 |
| **PC O-16:1;0/20:4;0** | 0.0359062408135414 | 0.0940162202597932 | -0.000128012555788559 | -0.116252877097242 | 0.116839987306004 |
| **PC O-16:2;0/18:0;0** | 0.0129531925995892 | 0.0605529347730544 | -0.0666812758364324 | 0.0462999013334694 | 0.114699472845819 |
| **PC O-17:0;0/15:0;0** | 0.0534650159655873 | 0.0448478987618015 | 0.0695639687724356 | 0.0805795004530989 | 0.0357515548070366 |
| **PC O-17:0;0/17:1;0** | 0.054385428957647 | 0.0680090459834898 | 0.0357256991778575 | 0.0790063631280136 | -0.0375489385012385 |
| **PC O-18:0;0/14:0;0** | 0.00697043980697698 | 0.00488569881518934 | -0.0163475449807226 | 0.0733896300615323 | 0.153302904301344 |
| **PC O-18:0;0/16:1;0** | 0.00926182180534859 | 0.0503865450594643 | 0.0280114337626996 | 0.0144522503918642 | 0.0174665578125717 |
| **PC O-18:0;0/20:4;0** | 0.0400198047167426 | 0.0922808681721188 | -0.0614731437495149 | -0.0711684846211264 | 0.0281969344183715 |
| **PC O-18:1;0/16:0;0** | 0.0390130718354696 | 0.126003184075497 | -0.0559776536379524 | -0.016010625491559 | -0.0140865706252078 |
| **PC O-18:1;0/18:2;0** | 0.0321396237740943 | 0.134755385407732 | -0.114516263890893 | -0.0159223359834565 | -0.0124588799443168 |
| **PC O-18:1;0/20:3;0** | 0.0466803586455739 | 0.0904992859593831 | -0.112109442017804 | 0.0591152870531531 | -0.0347776608355594 |
| **PC O-18:1;0/20:4;0** | 0.0463564081886535 | 0.114111770281109 | -0.0944791206609373 | -0.0999529513299259 | 0.0829752925773759 |
| **PC O-18:2;0/16:0;0** | 0.0409647449410287 | 0.124435088464877 | -0.0927852297901477 | -0.000277427059700878 | -0.0101748685612506 |
| **PC O-18:2;0/18:1;0** | 0.0260915547655416 | 0.112888436786181 | -0.0789163003788925 | -0.00933647161895055 | -0.0251342167854034 |
| **PC O-18:2;0/18:2;0** | 0.0334203052484618 | 0.120006707162609 | -0.106321684852611 | 0.00127916048178507 | -0.00084275635130857 |
| **PC O-18:2;0/20:4;0** | 0.0315048714208212 | 0.0973035884735996 | -0.0340380806522049 | -0.087289296977472 | 0.0480446775089569 |
| **PE 16:0;0_18:2;0** | 0.0875650841350613 | -0.0134375215794726 | -0.0173282901285005 | 0.0975905513341908 | -0.0547420260339511 |
| **PE 16:0;0_20:4;0** | 0.0851652865167207 | -0.0323530025652285 | 0.0146321121584087 | 0.0434271481462244 | 0.0175092848865401 |
| **PE 18:0;0_18:2;0** | 0.111110560775759 | -0.0447169284974976 | -0.0832724186278409 | 0.0856152130547045 | -0.0813553925065899 |
| **PE 18:0;0_20:4;0** | 0.101841816564687 | -0.0415868435644497 | -0.0332701751215416 | -0.0185466115147514 | 0.039424610483524 |
| **PE 18:1;0_18:1;0** | 0.0611119021256632 | -0.0252059272175732 | -0.0703893788104514 | 0.0583590758640955 | -0.018313235731246 |
| **PE O-16:1;0/18:2;0** | 0.0272415598985311 | 0.0593815169418683 | -0.0844870189200716 | -0.0121925323244004 | -0.0249336590823724 |
| **PE O-16:1;0/20:4;0** | 0.0496028719866275 | 0.0695392047676362 | -0.0402710530421788 | -0.0982993786099395 | 0.0743225195019649 |
| **PE O-18:1;0/18:2;0** | 0.0494530361268577 | 0.0987707366073927 | -0.130119099855165 | 0.0132487542691215 | -0.0216913862385331 |
| **PE O-18:1;0/20:4;0** | 0.0513383837395889 | 0.0839187914081729 | -0.0655532805582513 | -0.0926681606503218 | 0.0721286530041569 |
| **PE O-18:2;0/18:1;0** | 0.0375430856718645 | 0.0998576891247775 | -0.0734335748306237 | -0.00600196360738031 | 0.00279322311937054 |
| **PE O-18:2;0/18:2;0** | 0.0420729311525151 | 0.10048267306813 | -0.123646566950298 | -0.0129399712917107 | 0.0335998478124373 |
| **PE O-18:2;0/20:4;0** | 0.0501332935745567 | 0.08340981645398 | -0.0244578453678837 | -0.101096882979 | 0.0766148589049542 |
| **PI 16:0;0_18:1;0** | 0.0761335752229548 | 0.0281222958612668 | 0.00845430810586565 | 0.0726257792251815 | -0.0635692094095429 |
| **PI 16:0;0_18:2;0** | 0.086436392442102 | -0.00818224563937885 | 0.0160440833693512 | 0.103576933741535 | -0.0686937878471099 |
| **PI 16:0;0_20:4;0** | 0.0747187722991396 | -0.0268286143374491 | 0.102152513387497 | 0.0825786942765807 | 0.00125315891252056 |
| **PI 18:0;0_18:1;0** | 0.0838458967329538 | 0.0388344287660153 | 0.0119971829719806 | 0.0572457914523245 | -0.0538531661899269 |
| **PI 18:0;0_18:2;0** | 0.0852703540164239 | 0.0160609061818816 | -0.0314717585328278 | 0.0531731195695186 | -0.0422242084319521 |
| **PI 18:0;0_20:3;0** | 0.0934609263359489 | 0.000958842382692922 | -0.0181827023653744 | 0.0931292866044875 | -0.0729285685660847 |
| **PI 18:0;0_20:4;0** | 0.100702234633771 | 0.0218813568742131 | 0.0260668629903093 | -0.0303549223370579 | 0.0357860431598284 |
| **PI 18:1;0_18:1;0** | 0.0585172690798425 | 0.0609350833714251 | -0.0516783266294567 | 0.0520650655548759 | -0.0544129194472906 |
| **PI 18:1;0_18:2;0** | 0.0514065991953429 | 0.0348821708611941 | -0.0599280615170424 | 0.0584591731619556 | 0.00253487952234882 |
| **PI 18:1;0_20:4;0** | 0.0859394408855765 | 0.0187548421305109 | 0.0193450428920904 | 0.078040667081079 | 0.0140113001217478 |
| **PI 18:2;0_18:2;0** | -0.0014407727159116 | 0.0487601585397365 | -0.0577775213997314 | 0.0103944873719184 | 0.0171894398574422 |
| **SM 32:1;2** | 0.0723592152875738 | 0.0977689144411439 | 0.0516812032227952 | 0.012431867054201 | -0.0638344340215488 |
| **SM 34:0;2** | 0.0386853048259208 | 0.0681298999332195 | 0.0644114890995169 | -0.0530842122229327 | -0.0209589060447363 |
| **SM 34:1;2** | 0.0598804294444452 | 0.13382313273573 | 0.0173844680127527 | -0.0643643878053355 | -0.0429076278809303 |
| **SM 34:2;2** | 0.0655530214130584 | 0.134616996093802 | -0.00290806258667457 | -0.0398707551238316 | -0.0228355385514759 |
| **SM 36:1;2** | 0.0578175258478349 | 0.0944708891201445 | 0.0336234990702817 | -0.0954586816004058 | -0.0480459340639183 |
| **SM 36:2;2** | 0.0516487376155071 | 0.107805470801194 | -0.0043932682706274 | -0.0784711869174598 | -0.0136421039737421 |
| **SM 38:1;2** | 0.0655549607635749 | 0.0879552345065772 | 0.0177710812696815 | -0.040191457826675 | -0.0858717070601225 |
| **SM 38:2;2** | 0.0538382482416121 | 0.117307980070864 | -0.0129157153179745 | -0.0464433961795982 | -0.0476840025559426 |
| **SM 40:1;2** | 0.0649985290587556 | 0.0748414743315513 | 0.000998950311467488 | -0.0451598916779693 | -0.0716398618327122 |
| **SM 40:2;2** | 0.0598446799135393 | 0.114483065488575 | 0.0167637710019788 | -0.0411804573889828 | -0.0614811919312699 |
| **SM 42:2;2** | 0.0467055807207911 | 0.106893656493356 | 0.0664428703038797 | -0.114592886055617 | -0.0250652453229531 |
| **TAG 46:1;0** | 0.0733624725571422 | -0.0818193751078559 | 0.0358144396562884 | 0.112982239267628 | -0.0346204011048143 |
| **TAG 46:2;0** | 0.0723990745639215 | -0.0770604986487779 | 0.0194140180204185 | 0.10500704744041 | -0.0276202177143258 |
| **TAG 48:0;0** | 0.0623717134668937 | -0.0912133533460514 | 0.0391249285202998 | 0.0694095664768756 | -0.0397039870762781 |
| **TAG 48:1;0** | 0.0909321719030165 | -0.11115897470812 | 0.0353465829153315 | 0.0882482968228772 | -0.0349765333693257 |
| **TAG 48:2;0** | 0.0952199533671106 | -0.107818372038982 | 0.0155910770747625 | 0.0876989979495177 | -0.0238532432217239 |
| **TAG 48:3;0** | 0.0887607199557995 | -0.0974887481083515 | -0.014118529486657 | 0.0713138786144666 | -0.014556843665136 |
| **TAG 49:1;0** | 0.0838528144676054 | -0.094081496589552 | 0.0204831834918123 | 0.0697727811687776 | -0.0423192703185083 |
| **TAG 49:2;0** | 0.086589072937686 | -0.0874436467489784 | 0.0128782537924725 | 0.049234583972836 | -0.0245413572257009 |
| **TAG 50:1;0** | 0.100262642069459 | -0.128601540248147 | 0.0056282580686173 | 0.0368298676184632 | -0.0436986946620753 |
| **TAG 50:2;0** | 0.110918669276351 | -0.13009603593988 | -0.00495027418873098 | 0.0222791855558782 | -0.0215324481017376 |
| **TAG 50:3;0** | 0.112027181542515 | -0.126263145606982 | -0.0418296600722223 | 0.0008448431202948 | -0.0034349359208484 |
| **TAG 50:4;0** | 0.104043859524298 | -0.115221580935877 | -0.0463011411329857 | 0.00866597655972028 | 0.00614591809674245 |
| **TAG 50:5;0** | 0.0956030983997275 | -0.102940504607669 | -0.00250775941818365 | 0.020663234302895 | 0.0234619633976174 |
| **TAG 51:1;0** | 0.08665217064274 | -0.102354623292824 | 0.00756659416481433 | 0.0534411677907803 | -0.0475433817648651 |
| **TAG 51:2;0** | 0.106398905197402 | -0.114262262006367 | -0.023603650300956 | -0.00122279963971333 | -0.0266833777571876 |
| **TAG 51:3;0** | 0.104215448129406 | -0.106897194468439 | -0.0544490801458724 | -0.0298792908433322 | -0.00471740131018347 |
| **TAG 51:4;0** | 0.095535067886142 | -0.0879571633257158 | -0.0489356946264908 | -0.0327495904223125 | 0.00726124383014643 |
| **TAG 52:2;0** | 0.102779043181823 | -0.118785548562763 | -0.0508789142965117 | -0.0475537436442163 | -0.0131020251292256 |
| **TAG 52:3;0** | 0.102053723409451 | -0.11347755302982 | -0.103819064694068 | -0.0855470020102566 | 0.0030891197533229 |
| **TAG 52:4;0** | 0.0992812777839858 | -0.105180327017151 | -0.111976488497216 | -0.0836801679558151 | 0.00951936433608354 |
| **TAG 52:5;0** | 0.0996084483904492 | -0.10581695666051 | -0.0746488803379717 | -0.0647392779232746 | 0.023147985326908 |
| **TAG 52:6;0** | 0.0982775093238613 | -0.10633513222376 | 0.0339263785203666 | -0.0368712706948516 | 0.0435717068184417 |
| **TAG 53:2;0** | 0.102731414639214 | -0.0997743690233237 | -0.0580128323254651 | -0.0352832845420812 | -0.0237028208858386 |
| **TAG 53:3;0** | 0.0989003449831217 | -0.0908129142189568 | -0.0921708794231493 | -0.0767325421284521 | 0.000914520164134578 |
| **TAG 53:4;0** | 0.0957008042069131 | -0.0877607749721387 | -0.0883861525090027 | -0.0779244222035169 | 0.0178109141664873 |
| **TAG 54:3;0** | 0.0917150856611845 | -0.0928036135822454 | -0.12321366462221 | -0.0771147427803624 | -0.00877365612198541 |
| **TAG 54:4;0** | 0.0922047306878205 | -0.0885152679077418 | -0.152291127259048 | -0.0914245563915319 | 0.00540573078022213 |
| **TAG 54:5;0** | 0.0937775101570498 | -0.09777683695864 | -0.122456401972291 | -0.08349519801283 | 0.0237868241319712 |
| **TAG 54:6;0** | 0.0940520021669542 | -0.11110058822999 | -0.0210094398702464 | -0.0967819453659406 | 0.0412355362270741 |
| **TAG 54:7;0** | 0.0784735458343312 | -0.0867349494885408 | 0.0685780523188727 | -0.0946136993651225 | 0.0535116927432081 |
| **TAG 56:3;0** | 0.0569809326594575 | -0.0586443610581485 | -0.0505733488331067 | -0.0441618178787138 | -0.0103632902678369 |
| **TAG 56:4;0** | 0.0828275788117716 | -0.0813964923550914 | -0.0944478116081297 | -0.06010241330272 | 0.00143319256236777 |
| **TAG 56:5;0** | 0.0970198911828589 | -0.0919662035390672 | -0.084152855136169 | -0.131600207335833 | 0.0327557747890563 |
| **TAG 56:6;0** | 0.0995876926327616 | -0.100912614811831 | -0.0285886466600211 | -0.121736950542742 | 0.0501356780040181 |
| **TAG 56:7;0** | 0.0705922576397942 | -0.0854349123438754 | 0.0854892845342766 | -0.151543114178888 | 0.0391037753195001 |
| **TAG 56:8;0** | 0.0569744563044022 | -0.0669200295229928 | 0.0742000313340049 | -0.149019616633593 | 0.0475183356341927 |
| **TAG 58:7;0** | 0.0817932082963464 | -0.0873645261176879 | 0.0610701741881934 | -0.117303215981078 | 0.0417260265606927 |
| **TAG 58:8;0** | 0.0514836580748144 | -0.0606660572775869 | 0.0765335894863015 | -0.168556209299228 | 0.03730528487966 |
| **TAG 58:9;0** | 0.0368671076975102 | -0.0411141534702016 | 0.0908862694303151 | -0.151627193362278 | 0.0661213962834404 |

**Table S5.** Loadings of protein PCs 1-5 (MHO vs MUO)

| **Protein** | **PC1** | **PC2** | **PC3** | **PC4** | **PC5** |
| --- | --- | --- | --- | --- | --- |
| **X.101_IL8** | 0.0977013614420038 | -0.0115328791019674 | -0.0864574926411805 | -0.0472218408811484 | 0.0676002871942153 |
| **X.102_VEGFA** | 0.108742278343854 | -0.00239530160007033 | -0.0832392835226357 | -0.00377317712336158 | 0.079379187367882 |
| **X.103_AM** | 0.0867866485176902 | 0.0937166086120697 | -0.012081031410985 | 0.0946570708691143 | 0.145109697564335 |
| **X.105_CD40L** | 0.0964614274005877 | -0.161993765915218 | -0.103975812296613 | 0.032304970215066 | -0.00994749789117552 |
| **X.106_GDF15** | 0.100230496849418 | 0.0919425185229349 | -0.0841635308644962 | -0.127722416213164 | 0.0761887524516784 |
| **X.107_PlGF** | 0.114436563554436 | 0.106027803453673 | -0.0534168545017663 | -0.0233543687694405 | -0.047203821461221 |
| **X.108_SELE** | 0.0897312398899122 | 0.0608066574656466 | -0.0699696355500964 | -0.229730597868529 | -0.0839133109746963 |
| **X.109_EGF** | 0.0908888556424109 | -0.159498982323266 | -0.112501516207575 | 0.0290509754996629 | 0.0015372497107561 |
| **X.110_OPG** | 0.110691546455618 | 0.0741583373084724 | -0.0423011961087422 | -0.0820755630126048 | 0.013999799592467 |
| **X.111_SRC** | 0.000479978944824392 | 0.00406412563428725 | 0.0650759876613292 | 0.0501315997151264 | -0.0347873174628091 |
| **X.112_IL1ra** | 0.0697784778635882 | 0.0428776831973015 | -0.0236451840875718 | -0.0968202386702459 | 0.232515817119825 |
| **X.113_IL6** | 0.0643287500098669 | 0.0523082783397243 | -0.0332070036592533 | -0.106092535819407 | 0.177855502987096 |
| **X.114_CSTB** | 0.114243289463551 | 0.0344479955655983 | -0.0766209697885478 | 0.0240393669511146 | 0.0966731622626879 |
| **X.115_MCP1** | 0.0903282947521303 | 0.101910084708653 | -0.0649321387877607 | 0.0193269865537696 | 0.00495254628713573 |
| **X.116_KLK6** | 0.0902073485804521 | 0.0867024480528384 | -0.0252238656740013 | 0.0640208749322524 | -0.153561849306713 |
| **X.117_Gal3** | 0.10109661621688 | 0.0939973532254153 | -0.0402577428283005 | 0.0886487597350502 | 0.0772333875508776 |
| **X.118_PAR1** | 0.112717731761067 | -0.0965617475297173 | -0.0781636667167496 | 0.0983512396542335 | -0.0437420696251015 |
| **X.120_TRAIL** | 0.0960129561049247 | 0.0672907199939327 | -0.0165146129733627 | 0.0513111647708796 | -0.0836572335358185 |
| **X.121_hK11** | 0.0959523730966679 | 0.080289089198447 | -0.025300623726327 | 0.0905262412996016 | -0.0503466672940446 |
| **X.122_TIE2** | 0.108315767137898 | 0.0680599933046205 | -0.0424212973863068 | -0.0299368783115183 | -0.137227304175521 |
| **X.123_TF** | 0.103086633748173 | 0.0913580184149455 | 0.00414310725799004 | 0.126767134622259 | -0.076687383573781 |
| **X.124_TNFR1** | 0.116538789043794 | 0.0902440090733981 | -0.0021484493933715 | 0.0510476625490463 | 0.058451940128394 |
| **X.125_PDGFsubunitB** | 0.0977233131004578 | -0.146661278358117 | -0.107048833326343 | 0.0184891217596413 | 0.01063155803026 |
| **X.126_IL27A** | 0.0905873972093847 | 0.0853458388679224 | -0.0076884921378811 | 0.0928547123208388 | -0.000914871420105234 |
| **X.127_CSF1** | 0.107887113985563 | 0.0841992110795824 | -0.00721731157211836 | 0.118772283256379 | 0.029452230258488 |
| **X.128_CXCL1** | 0.0837284192736057 | -0.13506064897423 | -0.0875391449961093 | 0.0491147253405941 | 0.014568376445335 |
| **X.129_LOX1** | 0.0707759971863517 | 0.0525097732758171 | -0.0184745864644642 | -0.000380334474821804 | 0.121596885082784 |
| **X.130_TRAILR2** | 0.105922517275199 | 0.0889364569418565 | -0.0371302416392197 | -0.0159186194325797 | 0.101030798662065 |
| **X.131_FGF23** | 0.0864448342705696 | 0.053482509749846 | -0.0593200318494239 | 0.0480750245550903 | 0.0674920191980386 |
| **X.132_SCF** | 0.0601753973462417 | 0.061647619943715 | 0.0756309757189803 | 0.271572483791383 | -0.13953545250452 |
| **X.133_IL18** | 0.094831022900195 | 0.0517549796769578 | -0.0518693386328169 | -0.112297472123776 | -0.0210871787269719 |
| **X.134_IL6RA** | 0.0914942188786571 | 0.0715698509008426 | -0.0657189792490644 | -0.0173059114845312 | -0.0439686085981591 |
| **X.135_TNFR2** | 0.122739152638931 | 0.124619225830639 | -0.0299477308182668 | 0.0490234332118213 | 0.0693965816175093 |
| **X.136_MMP3** | 0.0649827752837993 | 0.0639227086955756 | -0.0617239390407813 | -0.0680655719283515 | -0.187226201087478 |
| **X.137_HSP27** | 0.0938750255228128 | -0.154672522986443 | -0.111326169383446 | 0.0543789039924485 | 0.000541986330811014 |
| **X.138_TNFSF14** | 0.107496606385589 | -0.092930391973464 | -0.097077277013237 | 0.0375661859332811 | 0.0541668675816638 |
| **X.139_PRL** | 0.0468688707565358 | 0.0696485995539503 | -0.0116781361867504 | 0.105095708662106 | -0.0598564185370742 |
| **X.140_MPO** | 0.0854574120917683 | 0.0694016842473681 | -0.053047914525145 | 0.0432216077480572 | 0.0334144934419731 |
| **X.141_GH** | 0.0149270852708422 | 0.0132463242357052 | 0.0272581619830647 | 0.165404642176218 | 0.140362989922492 |
| **X.142_MMP1** | 0.0525764158785155 | -0.0751239290530969 | -0.0504961121405402 | 0.0917133847998233 | 0.0622397681183188 |
| **X.143_RETN** | 0.0854262648319574 | 0.0708645321825573 | -0.0481316012541333 | 0.0377140513140777 | 0.0499916288569557 |
| **X.144_FAS** | 0.110913365345362 | 0.0968696973701776 | -0.0639569450688994 | -0.023500713723167 | -0.0578264295073629 |
| **X.145_PAPPA** | 0.0824084732904957 | 0.0553247055015976 | -0.111219211091193 | -0.0517717844236146 | -0.208412968798326 |
| **X.148_PTX3** | 0.052649966814623 | 0.0712325653029573 | -0.0216617087384552 | -0.0131713945256267 | -0.0307966873956762 |
| **X.149_REN** | 0.0688227087894957 | 0.0482844434799718 | -0.0509944891154368 | -0.153717650204495 | -0.0201836428462491 |
| **X.150_CHI3L1** | 0.0816708318371473 | 0.0807030493186893 | -0.0440251272188868 | -0.131280027285651 | 0.0268148819771438 |
| **X.151_ST2** | 0.0747791613178306 | 0.0404208825159334 | -0.0718022188108543 | -0.136467428652912 | -0.141010615860404 |
| **X.152_TIM** | 0.0804395368595149 | 0.0571420345475372 | -0.00840590209483455 | -0.145147044660375 | 0.0703450286029146 |
| **X.154_mAmP** | 0.0163909894973252 | 0.0179202354522473 | -0.0600666191181844 | -0.00506161719616946 | -0.0923633997416918 |
| **X.155_TRANCE** | 0.0589416051438511 | 0.0627217927849859 | -0.00918073494604312 | 0.0751884084258908 | -0.086288359171864 |
| **X.156_HGF** | 0.116419202297023 | 0.0387226853322557 | -0.0620469248747279 | -0.121007773536694 | 0.0466904006642315 |
| **X.157_PSGL1** | 0.0512219916041147 | 0.0224046459811001 | -0.0173445944960791 | 0.0993798942559073 | -0.0389850642885692 |
| **X.158_MB** | 0.0754260184420381 | 0.0702944004779683 | -0.0491075706039133 | -0.00864714032750083 | -0.172859997167876 |
| **X.159_TM** | 0.10878797596877 | 0.0602583462555378 | 0.00135116995976303 | 0.0714540245501485 | -0.0780366620610152 |
| **X.160_IL16** | 0.0981416719229179 | 0.047923699281526 | -0.00524901816976475 | 0.0671472452905091 | -0.0213548377224223 |
| **X.161_MMP10** | 0.0642186340074028 | 0.0500643645375328 | 0.0477371511812365 | 0.0660077354865759 | 0.0724352071214106 |
| **X.162_UPAR** | 0.104995272413826 | 0.0819066073232288 | -0.0193312873170273 | 0.0601301486274086 | 0.0951728411509254 |
| **X.163_CCL4** | 0.0841530471299138 | 0.00638354454600229 | -0.0701275486642259 | -0.0545890754018913 | 0.0217698108144157 |
| **X.164_CTSD** | 0.103270470267544 | 0.0253795505119563 | -0.0699987215973483 | -0.229969165501756 | 0.00204735040336648 |
| **X.165_RAGE** | 0.0814650257318685 | 0.0791634379191211 | -0.0149095285867395 | 0.192614612550641 | -0.0725973857093113 |
| **X.166_CCL3** | 0.0932759981920865 | 0.0721032107832087 | -0.043156119400169 | -0.0435025578718548 | 0.0863765882977881 |
| **X.167_MMP7** | 0.0935092834876428 | 0.024062322522307 | -0.0588976076027667 | -0.0586921668379752 | 0.0717386860203756 |
| **X.168_CXCL6** | 0.0910749482909461 | -0.105504273450281 | -0.0955757204931313 | 0.0559214902615845 | -0.0157700457954334 |
| **X.169_ITGB1BP2** | 0.0959465093013816 | -0.18612867883252 | -0.0938794448390973 | 0.0453854493010725 | 0.0139391166389465 |
| **X.170_CXCL16** | 0.108899429548017 | 0.0965675543080218 | -0.101569126992565 | 0.0118535545279658 | -0.0323858731746649 |
| **X.171_Dkk1** | 0.107587331756321 | -0.106625859641579 | -0.100015032664378 | 0.0676392535045161 | 0.00867697120297843 |
| **X.172_SIRT2** | 0.0990569358910102 | -0.185931834452278 | -0.101446770584747 | 0.049347303678355 | -0.000479440237276182 |
| **X.173_GAL** | 0.0593830873330315 | 0.0395100873338651 | -0.0175928506868247 | -0.0309359441962311 | -0.21782832387881 |
| **X.174_AGRP** | 0.0978833722084775 | 0.0820034356507854 | -0.013234354083016 | -0.00335674668410339 | -0.115149914974218 |
| **X.176_CD40** | 0.100109974753396 | -0.0929514064051271 | -0.073471886616932 | 0.0983221464124845 | 0.0427778304480093 |
| **X.177_tPA** | 0.0700952678648996 | 0.0157112846302816 | -0.0512036684805272 | -0.0862031123405037 | -0.053611861857126 |
| **X.178_HBEGF** | 0.112203283848579 | -0.115279550597018 | -0.0861521598609333 | 0.0887461842762999 | -0.0113942421727625 |
| **X.179_ESM1** | 0.0896842275817043 | 0.0961814301944801 | -0.0681992605631653 | 0.0732697250549719 | -0.12908851267588 |
| **X.181_VEGFD** | 0.0615544085891845 | 0.0666385565295723 | -0.0203844063707905 | 0.16626362734361 | -0.0934773502121407 |
| **X.182_MMP12** | 0.076741346096318 | 0.0852404540298532 | -0.0211951966767867 | -0.0991375104994262 | 0.0925843116061506 |
| **X.183_SPON1** | 0.10899059232254 | 0.096358646691211 | -0.0754095711416862 | 0.013302737315085 | -0.0360883420600861 |
| **X.184_CASP8** | 0.0999660803439534 | -0.0743257779619483 | -0.0411838445076505 | -0.0238350596008451 | -0.0625382647349113 |
| **X.185_CTSL1** | 0.102008803659841 | 0.0773512753953517 | -0.0361967700317103 | -0.0084058052448123 | -0.0667607486587754 |
| **X.186_CX3CL1** | 0.0858639284421625 | 0.0856514387193798 | 0.0113447149881232 | 0.195939953697226 | -0.0163755142597732 |
| **X.187_FABP4** | 0.0761040709751134 | 0.0782261193869313 | -0.0053897213714616 | 0.122792424848052 | 0.197744804894097 |
| **X.189_LEP** | 0.0287616574039469 | 0.013588235582281 | 0.058239457829041 | 0.165676228952408 | 0.212825704670248 |
| **X.190_CCL20** | 0.0627622383257302 | 0.0319342118915509 | -0.0286227886638232 | -0.152462804900091 | 0.130483418193348 |
| **X.191_CA125** | 0.0576798486773656 | 0.0478262512418747 | -0.0694372731800454 | 0.00323318048361036 | -0.0818124236895633 |
| **X.192_NEMO** | 0.0647166387720104 | -0.169126415819902 | -0.0577127561368653 | 0.0336248565822994 | -0.00671286455769218 |
| **X.193_FS** | 0.0950678100350638 | 0.052159910350295 | -0.0643174800376497 | -0.0762125752072428 | 0.038701465307126 |
| **X.194_PECAM1** | 0.11545687613248 | -0.0585713955040539 | -0.0870083968800155 | 0.000355219706733173 | -0.0849956468557565 |
| **X.195_NTproBNP** | 0.0294009471490792 | 0.0666743692920311 | 0.0164459296879711 | 0.0508102508206006 | 0.11144769693069 |
| **X.196_ECP** | 0.0576160054112454 | 0.0498308368833083 | -0.0160615810395335 | 0.0669093288084222 | 0.0810800272975753 |
| **X.O_110_ILT3** | 0.0844275466588292 | 0.0214740157343129 | 0.122779409025385 | -0.000617226452138723 | 0.0757785373836094 |
| **X.O_111_EZR** | 0.0772470202300544 | -0.0625320866958826 | 0.171942705063018 | -0.0885358040540455 | 0.0226892484754668 |
| **X.O_120_LAPTGFbeta1** | 0.110824728685067 | -0.142285441600498 | 0.0505510841010917 | -0.0555601946095821 | 0.00555690301614397 |
| **X.O_126_PARK7** | 0.0833911437950987 | -0.174231347393152 | 0.0259741158380586 | -0.000525176261739166 | -0.00633425525004343 |
| **X.O_128_CXCL11** | 0.0764691414553004 | -0.0934995433941479 | 0.0255197166608484 | 0.050355315253615 | 0.0952404522301513 |
| **X.O_129_IL12** | 0.0719433307947615 | 0.0255026360668292 | 0.136309988483241 | 0.0296602518102661 | 0.0966617415389301 |
| **X.O_131_IL7** | 0.0998379960272709 | -0.176960035930994 | 0.01256272286484 | -0.0172615704881429 | 0.0247887931846558 |
| **X.O_133_CXCL9** | 0.0777347597346274 | 0.0240283353317739 | 0.0999203231722554 | -0.016904391916268 | 0.13114330065256 |
| **X.O_137_FADD** | 0.0940482900136475 | -0.163725506971565 | 0.00662127577087454 | -0.00959386646078862 | -0.0487531290826893 |
| **X.O_140_FUR** | 0.0892383777204547 | -0.0489369026326439 | 0.106157647960451 | -0.148486149697798 | 0.0900317233900824 |
| **X.O_142_FasL** | 0.0622855029758083 | -0.00228465323112934 | 0.159232099290046 | 0.0855130627022645 | -0.035092773472757 |
| **X.O_143_BAFF** | 0.0826170355086173 | 0.0122018543189497 | 0.117338308847768 | -0.0588920305558507 | 0.0101726243084002 |
| **X.O_145_CCL19** | 0.0643725819491988 | 0.0216959156629902 | 0.0792736181809411 | -0.0838622448950679 | 0.101466684184598 |
| **X.O_149_EMMPRIN** | 0.0772843833875619 | -0.0284897369652422 | 0.208285358218101 | 0.14568125348184 | -0.0126227912950984 |
| **X.O_150_CAIX** | 0.0621088987685801 | 0.0028548843319499 | 0.0511625914686473 | -0.0244729345478104 | 0.0166115454590925 |
| **X.O_151_CXCL10** | 0.0752344151061577 | 0.00730955170954232 | 0.0682224773703196 | -0.0618493301169389 | 0.122506051385667 |
| **X.O_152_EpCAM** | 0.0163517430730784 | -0.0085990276345143 | 0.0703259344798479 | 0.0451526409501354 | 0.0295352568267851 |
| **X.O_153_ErbB2HER2** | 0.0855012810528982 | -0.0263083993757223 | 0.13277774233761 | -0.173144592230837 | -0.0986074093154729 |
| **X.O_154_ErbB3HER3** | 0.0823847981375182 | -0.0398356064704108 | 0.193481196499935 | -0.0269106469676597 | -0.0220883009496439 |
| **X.O_155_ErbB4HER4** | 0.0822436795418495 | -0.0293391148794381 | 0.155628635613807 | -0.0452960676846361 | -0.133699849493808 |
| **X.O_159_MIA** | 0.0826431196321167 | -0.0087583870903765 | 0.135316655985304 | -0.0339203860971751 | -0.0462598899001888 |
| **X.O_160_CXCL5** | 0.0818368535400203 | -0.176975323323196 | -0.0136026981023414 | 0.023361594107997 | 0.00163439666134516 |
| **X.O_161_MK** | 0.0963780508368435 | -0.0729644348313786 | 0.054772936760626 | 0.00579310880409086 | 0.0502674140883772 |
| **X.O_164_ICOSLG** | 0.0654952207325277 | -0.0042242803294971 | 0.217152139901354 | 0.0362986096115286 | -0.0762024359735852 |
| **X.O_165_CDH3** | 0.0832043714087203 | 0.0281522131777152 | 0.170513198112079 | 0.0528246285153332 | -0.0629528318553779 |
| **X.O_166_LYN** | 0.0781267510483194 | -0.193474062718999 | 0.0269500196478847 | -0.00248534969021222 | -0.00636825598716619 |
| **X.O_167_Flt3L** | 0.0782654728627227 | 0.000285804552098412 | 0.150652699765191 | -0.0036792767982418 | 0.00371752129944191 |
| **X.O_168_VEGFR2** | 0.0788726491999395 | -0.0403678159769188 | 0.161508599759242 | -0.0579156233059364 | -0.0668154554260547 |
| **X.O_170_CASP3** | 0.0965208323665368 | -0.201471479489443 | -0.0385983558153205 | -0.0232303939059926 | -0.0233885853348458 |
| **X.O_171_CD69** | 0.0774506111298156 | -0.200948384976409 | -0.0532360514943855 | 0.0242188894065166 | 0.000773323101043808 |
| **X.O_172_TNFRSF4** | 0.101885663500987 | 0.0318790187825653 | 0.163263423501892 | 0.0247108708199858 | 0.0941384281777808 |
| **X.O_173_TRAP** | 0.0853168896379344 | -0.0100496678555266 | 0.123209033967582 | -0.188542241888972 | -0.0954158610786867 |
| **X.O_174_CDKN1A** | 0.0862278639129003 | -0.207239503861185 | -0.0263518503371696 | -0.039983163013131 | -0.0330367339299959 |
| **X.O_175_REG4** | 0.0886726761287082 | -0.00228228803703802 | 0.106807937718127 | -0.0460379352940174 | -0.0146410046678167 |
| **X.O_176_TGFalpha** | 0.0973014070356986 | 0.0181538294222145 | 0.178272120730686 | -0.0378570111062738 | 0.0679861862851119 |
| **X.O_177_AR** | 0.0686519763504642 | -0.00280502092979014 | 0.102662839545382 | -0.0215826808618512 | 0.0571337616447938 |
| **X.O_179_MICA** | 0.0538640077810362 | -0.0345268096017707 | 0.0520871628567124 | -0.0776338036015855 | 0.0721696288538743 |
| **X.O_182_HE4** | 0.0873113858040406 | 0.0392775937244886 | 0.104858320063933 | 0.0104774729596749 | 0.0914250345711388 |
| **X.O_183_CXCL13** | 0.0745285778828365 | -0.00277013706664681 | 0.0739829365755123 | -0.0816571353987045 | 0.068296282340392 |
| **X.O_184_EGFR** | 0.0729473658779667 | -0.0123393302664716 | 0.133516071356645 | -0.0672952831833805 | -0.108300057609487 |
| **X.O_185_ITGA1** | 0.0776669617066294 | -0.0130765481542861 | 0.174609129306437 | -0.0395638962450061 | -0.151876080914503 |
| **X.O_186_THPO** | 0.086497946312537 | -0.0964802948102927 | 0.108947241103141 | 0.0604461775028822 | 0.0304109624045984 |
| **X.O_187_eIF4B** | 0.0826972627779136 | -0.189192736962076 | 0.0261242844306408 | -0.0034427109272495 | -0.0178720031953854 |
| **X.O_189_VIM** | 0.0581145835874003 | -0.0335790642657242 | 0.0825967818212158 | 0.0419458119529701 | 0.0942391093948632 |
| **X.O_190_NTRK3** | 0.0740932724441957 | -0.0164686047551544 | 0.197996230577358 | 0.0245328291256587 | -0.170307350889667 |
| **X.O_192_PRSS8** | 0.0727608323956667 | -0.0103422594184469 | 0.0929453317059149 | -0.148909420788952 | 0.012259901352981 |
| **X.O_195_IL17RB** | 0.0556299577662712 | 0.0233281835415006 | 0.0917555392397254 | 0.0579325607245841 | -0.0608149335489315 |
| **X.O_196_FRalpha** | 0.0977777450549218 | -0.00735707289585461 | 0.166556913216798 | 0.100354358176132 | 0.0065495663868676 |

**Table S6.** Correlation between biomarker PCs (PL2, PP5) and cardiometabolic risk factors.

|  | **PL2** |  | **PP5** |  |
| --- | --- | --- | --- | --- |
| **Risk factor** | **p-value** | **rho** | **p-value** | **rho** |
| **Antihypertensive treatment** | 0,004483 | -0,13942 | 0,000328 | 0,1757 |
| **BMI** | 0,00048 | -0,17086 | 2,59E-06 | 0,228645 |
| **CRP** | 0,105498 | -0,07967 | 3,78E-14 | 0,360477 |
| **Current Smoker** | 0,513237 | -0,03222 | 0,023256 | 0,111515 |
| **DBP** | 0,087893 | -0,08398 | 0,130221 | 0,074493 |
| **Glucose** | 2,16E-11 | -0,32123 | 0,011174 | 0,124588 |
| **Hba1c** | 4,78E-05 | -0,19845 | 0,002455 | 0,148482 |
| **HDL cholesterol** | 2,76E-40 | 0,590625 | 2,06E-08 | -0,27122 |
| **Homa_IR** | 6,7E-14 | -0,35715 | 1,15E-05 | 0,213748 |
| **LDL cholesterol** | 1,6E-05 | 0,210271 | 0,431507 | -0,03876 |
| **SBP** | 0,281565 | -0,05305 | 0,453312 | 0,036955 |
| **TG** | 6,22E-56 | -0,67304 | 3,41E-06 | 0,225982 |
| **Waist** | 1,16E-07 | -0,25686 | 8,49E-08 | 0,259513 |

**Table S7.** Loadings of metabolite PCs 1-5 (MHO vs NOC)

| **Metabolites** | **PC1** | **PC2** | **PC3** | **PC4** | **PC5** |
| --- | --- | --- | --- | --- | --- |
| **AAMU** | -0.0541616461396208 | -0.0105884869592304 | -0.00891454004538022 | -0.221396584501145 | -0.108825811063391 |
| **Acetylarginine** | -0.0700540153182414 | -0.0829638715517473 | -0.0446274554433706 | -0.0229774799349012 | 0.0283402172716519 |
| **Acetylcarnitine** | -0.122025001249116 | 0.11916329640721 | -0.0824058622392812 | -0.0410350775497965 | 0.0697384601508027 |
| **Acetylornithine** | -0.0531462085971753 | -0.00928829627728332 | 0.0396116547273414 | -0.126696878387637 | 0.0660105078205672 |
| **Acisoga** | -0.0874413278030443 | -0.00256152650926865 | -0.016031668701523 | -0.142445552947623 | 0.0495319708851876 |
| **ADMA** | -0.0849755513526275 | -0.0417032066010303 | -0.0729112831597439 | -0.0593445896459536 | 0.0183467295263422 |
| **Alanine** | -0.0892569647359737 | -0.155008692909075 | -0.0524096077000212 | 0.0786238833194201 | 0.0294994148451239 |
| **Arginine** | -0.0926831108328327 | -0.112893994834431 | -0.109033168870041 | -0.00106806269196228 | -0.058710040814194 |
| **Asparagine** | -0.124053979003071 | -0.133936509386 | -0.200527610224695 | 0.0273420851293471 | -0.106985689982005 |
| **Beta.carotene** | -0.0353416331063746 | -0.00666633370247394 | -0.0459615051517267 | -0.0481730667314288 | -0.0637711698096004 |
| **Betaine** | -0.109909767318776 | -0.0293521490625977 | -0.00882642976403174 | 0.0570142945671567 | -0.0540905068525572 |
| **Butyrylcarnitine** | -0.0666314233291686 | -0.0263584337526773 | 0.0666393483228704 | -0.0508279052889326 | 0.0964736337635977 |
| **C10.0.carnitine** | -0.108255511904749 | 0.177556156366945 | -0.0113177767498324 | 0.0606719539423608 | -0.0107438180757276 |
| **C10.0.OH.carnitine** | -0.13403246028215 | 0.18231406603366 | 0.0689345446476918 | 0.0587251410576464 | -0.0366581501697697 |
| **C10.1.carnitine** | -0.125671465491744 | 0.166339719374424 | 0.0201039720121675 | 0.0309474625092324 | -0.00228710645575298 |
| **C10.2.carnitine** | -0.127565569689761 | 0.10055197884231 | 0.0904092946610077 | -0.0148816646183883 | 0.020987433198657 |
| **C10.3.Carnitine** | -0.0906595219111596 | 0.0306307080202829 | 0.0655004314676983 | -0.0876360686801183 | -0.00264269495087293 |
| **C11.0.carnitine** | -0.131251572716913 | 0.13825321064351 | 0.0408245895544533 | 0.063779973534273 | -0.0162770995933695 |
| **C11.1.carnitine** | -0.142027634629421 | 0.0981993279041917 | 0.0929497991767561 | -0.00844246064202892 | 0.0378731930150965 |
| **C12.0.carnitine** | -0.136220348129061 | 0.21956270329573 | -0.0149105572720605 | 0.0740775467910246 | -0.0173646071735325 |
| **C12.1.carnitine** | -0.133888343246746 | 0.22739579934843 | -0.0384675584460129 | 0.0357065976994043 | -0.00888792547893442 |
| **C12.2.carnitine** | -0.145038970578915 | 0.172927060105908 | 0.0474383465715277 | 0.0393643664870103 | -0.0170217408221256 |
| **C13.0.carnitine** | -0.0894701588226579 | 0.0662591819012641 | 0.0461282524217109 | -0.0144784584126249 | 0.031353490298566 |
| **C13.1.carnitine** | -0.118855037054629 | 0.102652111619366 | 0.0588812103123961 | 0.0177942933126116 | 0.0321535832070213 |
| **C14.1.carnitine** | -0.124073681541618 | 0.226863376942607 | -0.0642833641647565 | 0.0318626210183838 | -0.00929572077638748 |
| **C14.2.carnitine** | -0.124496930156392 | 0.206085406779416 | -0.0239418943218145 | 0.0443477090796245 | -0.0210394760480897 |
| **C16.1.carnitine** | -0.124576846058647 | 0.19089976681285 | -0.149659630849768 | -0.0255195985793434 | 0.0270288482211943 |
| **C18.0.carnitine** | -0.113765011091199 | 0.0644991721886392 | -0.123587637132443 | 0.00966103757229241 | 0.0198085664451286 |
| **C18.1.carnitine** | -0.147166511050098 | 0.121207420882484 | -0.165210259623877 | -0.00350811436379541 | 0.0306113538651853 |
| **C18.2.carnitine** | -0.144671538892826 | 0.109238697152806 | -0.145102308285062 | 0.0108160063664269 | 0.0200940046578433 |
| **C4.0.OH.carnitine** | -0.0101190747018314 | -0.0128214259598752 | 0.00262193107685944 | 0.0365794736062435 | -0.0157389098961741 |
| **C6.0.carnitine** | -0.103446827993927 | 0.149948215302328 | -0.00141549493602612 | 0.0177685290289877 | 0.0284127588881171 |
| **C8.0.OH.carnitine** | -0.120349023647473 | 0.159326973175502 | 0.0547744893008207 | 0.0556362447801301 | -0.0520810901475451 |
| **C8.1.carnitine** | -0.107667223329204 | 0.0480517312562133 | 0.0785650332313909 | -0.0937805945293453 | 0.0298433619358907 |
| **C9.0.carnitine** | -0.0939382693555812 | 0.0504059481819729 | 0.0978502154007204 | 0.0102264560295082 | -0.00327736675787137 |
| **Caffeine** | -0.0184790306984222 | -0.00492219898776919 | -0.000275385439176166 | -0.0581873760729623 | -0.0547486524266159 |
| **Carnitine** | -0.0614056953720026 | -0.0363259876932138 | -0.00630362660764087 | 0.00418304361390378 | 0.0302585714381527 |
| **Choline** | -0.0747354109189265 | -0.0361798421913405 | -0.0740079532863535 | 0.0397965474948142 | 0.224818640330911 |
| **Citrulline** | -0.117531317702683 | -0.07175305517179 | -0.0883186153121115 | -0.107677642697112 | -0.0430842164990169 |
| **Cotinine** | -0.0402639478647622 | 0.0174966744804357 | -0.050987240136728 | -0.0332532642249876 | -0.089148076917185 |
| **Creatine** | -0.00776893596208585 | -0.0740119332291686 | -0.181319449729232 | -0.200166661867083 | 0.212377123163403 |
| **Creatinine** | -0.130546506147456 | -0.0608028584866097 | 0.233682095579047 | -0.0264409132755594 | -0.13990461257781 |
| **Cystine** | -0.127937090563625 | -0.0778041060442689 | 0.0224172399005071 | -0.0991937513550126 | -0.131954623846956 |
| **Dimethylglycine** | -0.0599886206110837 | -0.00466320925496695 | 0.0293277829331688 | -0.0369872046322849 | 0.0247115744727032 |
| **Dimethyllysine** | -0.0564220462976217 | -0.026570635363958 | -0.025103737261375 | -0.16567422701204 | 0.050221390744847 |
| **DMGV** | -0.0494734285470483 | -0.0939800750858813 | 0.168766489746544 | -0.0596788075716734 | 0.0503757726606401 |
| **Ergothioneine** | -0.0312168742027285 | -0.0382346002534068 | 0.0199170380001231 | 0.0425364855663627 | 0.0548628155889867 |
| **Glutamate** | 0.0376015223395172 | 0.0251507111982667 | -0.0253850964226409 | 0.11744411676938 | 0.346605691289602 |
| **Glutamine** | -0.133042322773012 | -0.0923442729619653 | -0.106818573412384 | -0.0802334395883024 | -0.29783198749923 |
| **Glycerophosphocholine** | -0.0184717153185764 | -0.0351974669529001 | -0.122309313371882 | 0.0974642272056705 | 0.147670440476484 |
| **Guanidineacetate** | -0.0716082107756568 | -0.0264961906896965 | -0.018552786237933 | 0.111459376711575 | -0.237099411054895 |
| **Hippurate** | -0.0296451045251861 | -0.0214151143405348 | 0.0124701487682943 | -0.177306086166421 | -0.0484734500654256 |
| **Histidine** | -0.128787148614411 | -0.137001020740001 | -0.127890944507912 | 0.0650641510279161 | -0.0321770741936348 |
| **Homoarginine** | -0.048802681814285 | -0.0959888762194611 | 0.0424715000593873 | 0.168142567076868 | -0.0166904348255613 |
| **Homocitrulline** | -0.0369972953945884 | -0.0122764898166359 | 0.0766614233521788 | -0.133819977394799 | 0.144778086835525 |
| **Homostachydrine** | -0.0396834957135365 | 0.00489772757856484 | -0.0342771043071308 | -0.0522498774961253 | -0.00188382377855328 |
| **Hydroxycotinine** | 0.0292094058230301 | 0.0155409451716506 | 0.0333199469589163 | 0.10584898288066 | 0.221615489502473 |
| **Hypoxanthine** | -0.0395104331536605 | -0.0367723768585828 | -0.0630848154704481 | -0.0304571050577004 | 0.215380045620034 |
| **Isoleucine** | -0.124558856018281 | -0.146544540054525 | 0.103069768261759 | 0.196581121533985 | 0.0271674659605396 |
| **Isovalerylcarnitine** | -0.117785691637305 | -0.0517249700930875 | 0.110997986937314 | 0.0547871226446597 | 0.09895785595792 |
| **Kynurenate** | -0.100420563915854 | -0.0619648267445554 | 0.145772105918784 | -0.0284957488772872 | 0.0458995143866416 |
| **Kynurenine** | -0.117864179652619 | -0.0930369060871306 | 0.136111582576934 | -0.112191013591701 | 0.0419799289066488 |
| **Leucine** | -0.133507838272232 | -0.148365589735245 | 0.075543421098664 | 0.213183642439227 | 0.0280940121535149 |
| **Lysine** | -0.136819911912123 | -0.149521068606192 | -0.121713333770116 | 0.0598470285129732 | 0.0399174528860984 |
| **Methionine** | -0.139845844848902 | -0.151890031717139 | -0.0815786894860935 | 0.128674025199722 | -0.0576151902476567 |
| **Methionine.S.oxide** | -0.022979183165997 | -0.036116182709063 | -0.0333400376091715 | 0.0079319347378547 | 0.0632510502875833 |
| **Methyllysine** | -0.0364371546925433 | -0.0080639126859485 | -0.0566108660250444 | -0.152927942142015 | 0.041326064215851 |
| **Methylnicotinamide** | -0.0632090184086762 | -0.0265258096596633 | -0.0135355682068761 | -0.0160246914256159 | 0.0793976290406954 |
| **Myristoylcarnitine** | -0.142263167874392 | 0.168112220917272 | -0.0681345003309036 | 0.0597783690867585 | 0.0209052820075477 |
| **N.Acetylcarnosine** | -0.106718525936098 | -0.0453867727041143 | 0.232239161387344 | 0.178474787498902 | -0.0575893605885654 |
| **N.Methyl.2.pyridone.5.carboxamide** | -0.0529534084955112 | -0.0161356432444123 | 0.0836409400848616 | -0.0870789783187377 | 0.12221206192952 |
| **N.Methyl.4.pyridone.3.carboxamide** | -0.060414383542141 | -0.0254342456830254 | 0.0837034724669991 | -0.0982483051532801 | 0.114949826141007 |
| **N.Methylproline** | -0.011405360873731 | -0.0173620938985607 | -0.0083375505494169 | -0.0784830248393315 | 0.0398808708475248 |
| **N2.N2.Dimethylguanosine** | -0.109304698961841 | -0.0486104446715659 | 0.113852586967784 | -0.151372009324413 | -0.0804088019163639 |
| **Nicotinamide** | -0.0704579004036443 | -0.0678423034568287 | -0.0648970775768661 | 0.0537239718117652 | 0.165095505297116 |
| **NMMA** | -0.0637863801162047 | -0.0789257615238156 | -0.146431750050128 | -0.0982537106550201 | 0.00274289230836593 |
| **Octanoylcarnitine** | -0.0982831153244255 | 0.151652463991869 | -0.0171523891306147 | 0.0558103217000669 | -0.00115071996899818 |
| **Ornithine** | -0.111358855978739 | -0.0927260848555036 | -0.146652438522387 | -0.00176846743467388 | 0.0172662614209071 |
| **Palmitoylcarnitine** | -0.155067173896022 | 0.0829471052019388 | -0.105230708607735 | 0.0751921150489222 | 0.0671118096843041 |
| **Pantothenate** | -0.0454692030285784 | -0.0133464485709989 | -0.00661881486174438 | -0.0501065611263001 | 0.103729426525456 |
| **Paraxanthine** | -0.0451588198290042 | -0.009136805028421 | -0.0132695379915891 | -0.118475977788337 | -0.0817699747042447 |
| **Phenylacetylglutamine** | -0.0540827518951417 | -0.0364842912271647 | 0.065656271734694 | -0.138578826765186 | 0.0107269881542266 |
| **Phenylalanine** | -0.154732956066119 | -0.140764400852058 | 0.00642346917047172 | 0.0851709574220654 | 0.0133756650668855 |
| **Pipecolate** | -0.017677020049842 | -0.0324369263865555 | -0.00195905274330349 | 0.0114493499252999 | 0.0206520021004623 |
| **Piperine** | -0.0118331656293861 | -0.0368873706612366 | 0.0758683200366508 | 0.0523462123445224 | 0.0295228619927221 |
| **Proline** | -0.0812892218871329 | -0.134196846602902 | 0.000191318215763914 | 0.11668143156468 | -0.0524398938113567 |
| **Proline.betaine** | -0.0122803175908098 | -0.00444744030447388 | 0.00110762938443507 | -0.0973892340254456 | 0.0482180920612152 |
| **Propionylcarnitine** | -0.10252796152375 | -0.0822509650949233 | 0.0936790581855467 | 0.0130146621999923 | 0.136772377519368 |
| **Pyroglutamate** | -0.0863160565137795 | -0.0481313968347267 | -0.115414766783154 | 0.0362830765299351 | -0.0225329887318041 |
| **Sarcosine** | 0.0121372903254385 | -0.03949620013002 | -0.103498930442478 | -0.114511443736955 | 0.15469368133653 |
| **SDMA** | -0.111899224286627 | -0.0504523608933839 | -0.0668316683112467 | -0.140039912231355 | 0.0209282316241482 |
| **Serine** | -0.0857370776816323 | -0.0703319409876475 | -0.308363722902228 | -0.0668899489350586 | -0.0675348969836119 |
| **Taurine** | -0.0973586450752338 | -0.066126726002313 | -0.134776623616229 | -0.0945554088516538 | 0.117064533530037 |
| **Threonine** | -0.0795188580238361 | -0.123196370516165 | -0.150755985977491 | 0.0630166244215121 | -0.0491974919011985 |
| **Tiglylcarnitine** | -0.0895345425950792 | -0.0103055750067511 | 0.138451982884489 | -0.0907789520714935 | 0.0405005318869426 |
| **Trigonelline** | -0.0033467350595191 | 0.0182817029552831 | -0.0217036463539333 | -0.225712127626837 | -0.0609819437665172 |
| **Trimethylamine.N.oxide** | -0.0248471631168978 | -0.018558216884166 | 0.0591521567480569 | -0.0352398548368962 | 0.0427212771361355 |
| **Trimethyllysine** | -0.0743996932913822 | -0.0596948562290187 | 0.104809578741182 | -0.0193989657238821 | 0.0333509703070237 |
| **Tryptophan** | -0.117133559865739 | -0.164208739529677 | -0.0415404207753044 | 0.156971444331909 | 0.0220724777884272 |
| **Tyrosine** | -0.075597907050853 | -0.102981772697023 | 0.0027309911004029 | 0.0638304714419777 | 0.0134972329567278 |
| **Urea** | -0.10133970748024 | -0.0802988870493865 | 0.127920312349081 | -0.0365750873049435 | 0.105577984564259 |
| **Urocanate** | -0.0182237169849128 | -0.00831759953352719 | 0.0103391135575799 | -0.0368526430011729 | 0.00964513719877321 |
| **Valine** | -0.120043022400809 | -0.142169100469692 | 0.0819400023933996 | 0.160939955483675 | 0.0390290826040545 |
| **X1.Methyladenosine** | -0.10276267103857 | 0.00968504108686844 | 0.0553366834426327 | -0.241240264304064 | 0.00544079824529782 |
| **X1.Methylhistidine** | -0.120372673498277 | -0.0432974931302931 | 0.135092120987521 | -0.113759318382567 | 0.101847412705626 |
| **X2.Aminoisobutyrate** | -0.0855359836277696 | -0.0178614808628436 | -0.0392715600024227 | 0.0854305114605838 | 0.0833929429903707 |
| **X25.hydroxyvitamin.D3** | -0.0356310010895878 | -0.00594248945374294 | -0.00391052291203002 | -0.00474788783096739 | 0.0402345556152232 |
| **X3.Hydroxytrimethyllysine** | -0.11648487882924 | -0.0674638412411302 | 0.109926679180791 | -0.0987248468977977 | 0.0405200987477758 |
| **X3.Methylhistidine** | -0.0217305601246879 | -0.0252689091498843 | 0.0642829487839301 | -0.0228161762579179 | 0.0709446830219694 |
| **X4.Trimethylammoniobutanoate** | -0.11919945243467 | -0.0556136057099346 | 0.188014966732656 | 0.0350876733467127 | -0.0897473310592898 |
| **X5.Methylthioadenosine** | -0.110898268006532 | -0.0712275925663386 | -0.0802484618564069 | -0.0934465005150584 | -0.312965378535561 |
| **X7.Methylguanine** | -0.105859033320441 | -0.0271208067065609 | 0.145962959278595 | -0.145753284850982 | -0.146269508605328 |

**Table S8.** Loadings of lipid PCs 1-5 (MHO vs NOC)

| **Lipids** | **PC1** | **PC2** | **PC3** | **PC4** | **PC5** |
| --- | --- | --- | --- | --- | --- |
| **CE 14:0;0** | 0.0964229400108342 | -0.0176931987379625 | -0.0585027385023744 | 0.142240735541634 | -0.0422007721726075 |
| **CE 15:0;0** | 0.0626659444785569 | 0.0218138353570167 | -0.0532737304519628 | 0.0548781418656257 | -0.0352575047681615 |
| **CE 16:0;0** | 0.114586943576888 | 0.0264019912031006 | -0.127747177638248 | -0.00716566686076648 | 0.00837852153947279 |
| **CE 16:1;0** | 0.08028827426726 | -0.0142336502416386 | -0.0709827689190067 | 0.169350461795425 | -0.019089929870404 |
| **CE 17:0;0** | 0.0774765203862366 | 0.0401961142924982 | -0.0655292005295813 | 0.0117261467937766 | -0.0235837286955403 |
| **CE 17:1;0** | 0.100726392653642 | 0.0225263463538537 | -0.0939348428173596 | 0.119028105295492 | -0.0396192737452911 |
| **CE 18:0;0** | 0.0852945908993537 | 0.00408304571612908 | -0.0410171590332943 | -0.0838537970278758 | -0.00047727375338251 |
| **CE 18:1;0** | 0.112424182665487 | 0.0298471490232512 | -0.0942998099277906 | 0.0425411898385746 | -0.0118885151486294 |
| **CE 18:2;0** | 0.100438221347028 | 0.0667699455486222 | -0.0214209385839597 | -0.117756891512263 | -0.0586394198993313 |
| **CE 18:3;0** | 0.10452044346377 | -0.00691884243472493 | -0.0438482924243409 | 0.0966182321661309 | -0.025591770699592 |
| **CE 20:2;0** | 0.0533212005884995 | 0.0554398693634642 | 0.0206391635130052 | -0.0564885910592087 | -0.0210952610889922 |
| **CE 20:3;0** | 0.0904808417996792 | -0.0200790728781197 | -0.0589697474978119 | 0.0202590723758668 | -0.040721268967067 |
| **CE 20:4;0** | 0.0801360699471792 | 0.02856778506025 | -0.160059267224559 | -0.0537506093541435 | 0.081562906776876 |
| **CE 20:5;0** | 0.0424546804459485 | 0.000149626140525529 | -0.21582543533824 | 0.0225576108471224 | 0.0463460713960225 |
| **CE 22:6;0** | 0.0599521092573811 | 0.0198410286651353 | -0.212295059962556 | -0.0622830508219675 | 0.0313842018838722 |
| **Cer 40:1;2** | 0.091920222670918 | -0.0108789627381078 | 0.014731081556003 | -0.0599055878712383 | -0.0772188136549829 |
| **Cer 40:2;2** | 0.0802559671012766 | -0.000450630063577016 | 0.0106293720375066 | -0.070646558183787 | -0.0669398269961864 |
| **Cer 42:1;2** | 0.0966892285542919 | -0.000151757381451135 | -0.00274642969353394 | -0.0397195366436485 | -0.0535670894420295 |
| **Cer 42:2;2** | 0.101875895348459 | -0.00771633634315057 | -0.048838999251282 | -0.0721913172036856 | -0.0493517319296481 |
| **Chol** | 0.0445118739593303 | 0.0111476847059547 | -0.0229185940608766 | -0.0172249235025846 | -0.0291220628041252 |
| **DAG 16:0;0_18:1;0** | 0.0567850106693902 | -0.114531714774277 | 0.0320492473017034 | 0.0216298811985254 | 0.0509407535997954 |
| **DAG 16:0;0_18:2;0** | 0.0531909070886077 | -0.126110120557211 | 0.0448554647433556 | -0.0689760842322197 | 0.0560163677590055 |
| **DAG 16:1;0_18:1;0** | 0.0598743270792735 | -0.101140103739484 | 0.0146044561937188 | 0.0291259170619659 | 0.00820924713870442 |
| **DAG 18:1;0_18:1;0** | 0.0655739863506334 | -0.107936106661745 | 0.0328192266929751 | -0.0512112493395365 | 0.0177957869542747 |
| **DAG 18:1;0_18:2;0** | 0.0652757758380989 | -0.120160408517116 | 0.0500520550793984 | -0.150886480221352 | 0.0172304364321167 |
| **DAG 18:1;0_18:3;0** | 0.0492235504608327 | -0.0528679389675483 | 0.0137139677846891 | -0.0398453012375014 | 0.0151948964688742 |
| **LPC 14:0;0** | 0.0607364677261198 | -0.0344540183827332 | 0.0420388924597423 | 0.153814625247287 | 0.0882342714148004 |
| **LPC 16:0;0** | 0.0547788410011841 | -0.0156100151206789 | 0.051361848706037 | 0.0521587866140212 | 0.264517097608243 |
| **LPC 16:1;0** | 0.0594614278936902 | -0.0102769067872855 | 0.0594689898945235 | 0.097415295644953 | 0.180272566204823 |
| **LPC 18:0;0** | 0.0503768902907517 | 0.011219106847867 | 0.05431090752799 | -0.022396124561177 | 0.224520329837751 |
| **LPC 18:1;0** | 0.0531473783134021 | 0.0628467003964327 | 0.107562814797476 | 0.0408672019962471 | 0.232706128903389 |
| **LPC 18:2;0** | 0.0371905496090321 | 0.0801556957395262 | 0.123751812546485 | -0.00903604336955044 | 0.164278831407863 |
| **LPC 20:3;0** | 0.0593187823137462 | -0.00144033509114643 | 0.0722919217338313 | 0.0893648225322355 | 0.192267880107377 |
| **LPC 20:4;0** | 0.0404866755297025 | 0.0221592706475244 | -0.0173483691634771 | 0.0180183427714573 | 0.312544693108175 |
| **LPC 22:6;0** | 0.0316760537016075 | 0.0167688911138439 | -0.111087674188026 | -0.00138410904303382 | 0.205352358810249 |
| **LPE 16:0;0** | 0.077883411248315 | -0.00224537083508328 | 0.058235118761442 | 0.0956820553700673 | 0.139592979997969 |
| **LPE 18:0;0** | 0.0774996116553874 | -0.0243108810199057 | 0.0641112208972726 | 0.051097283327378 | 0.195157243016504 |
| **LPE 18:1;0** | 0.0576101378861514 | 0.0241415175582913 | 0.135621361396587 | 0.0664748595703414 | 0.10539800508368 |
| **LPE 18:2;0** | 0.0607509715628692 | 0.0397720432185487 | 0.174350451116157 | 0.0421106108045408 | 0.0355440764292732 |
| **LPE 20:4;0** | 0.0729003915990428 | 0.0366309437401635 | 0.0596584627545358 | 0.0268906183293333 | 0.136561169096104 |
| **PC 14:0;0_16:0;0** | 0.0709963378010576 | 0.0117006776871477 | -0.00945213602283167 | 0.174989333146801 | -0.052191150446982 |
| **PC 14:0;0_18:1;0** | 0.061155001052307 | 0.00403484416749573 | 0.0243113778704935 | 0.102417510327339 | -0.0422019327156747 |
| **PC 14:0;0_18:2;0** | 0.0753095425731138 | 0.0102392277680551 | 0.0311459594746958 | 0.0618193017608659 | -0.0840532750550009 |
| **PC 15:0;0_18:1;0** | 0.10024496826008 | 0.00838109828837661 | -0.0257300573672021 | 0.148395906495878 | -0.0325184463787081 |
| **PC 15:0;0_18:2;0** | 0.0998783476606946 | 0.0522880942674582 | 0.0565643805612469 | -0.00224573820378154 | -0.0972678794898833 |
| **PC 16:0;0_16:0;0** | 0.105470249944595 | 0.0519439242868374 | -0.0655466707691386 | 0.0690149677952316 | -0.0142289577976991 |
| **PC 16:0;0_16:1;0** | 0.0659802498807925 | -0.0126828575261231 | -0.0232126551185794 | 0.162560868346752 | -0.0452965924226093 |
| **PC 16:0;0_17:1;0** | 0.0515140507440125 | 0.0010090564363223 | -0.0198836058197702 | 0.0596630256719227 | -0.00682869294668024 |
| **PC 16:0;0_18:0;0** | 0.11000373592252 | 0.0537444235644177 | 0.0378839512482288 | 0.00129179670719164 | -0.0486852208033471 |
| **PC 16:0;0_18:1;0** | 0.103284641087142 | 0.0142355306964795 | -0.0274217080839503 | 0.145500488553598 | -0.02657803345481 |
| **PC 16:0;0_18:2;0** | 0.11246805466364 | 0.0612265481658468 | 0.0625234378926995 | -0.0198280604449124 | -0.0899390991708636 |
| **PC 16:0;0_18:3;0** | 0.0898374672215859 | 0.00759195879621551 | 0.00710006618225463 | 0.142373583604132 | -0.0600087670510131 |
| **PC 16:0;0_20:1;0** | 0.0684340735415252 | 0.0137606855646644 | -0.0845016079227277 | 0.0782726193675772 | -0.0140816866352336 |
| **PC 16:0;0_20:2;0** | 0.115705787675199 | 0.00442675077611653 | 0.0137223328261558 | 0.063542530211421 | -0.0437696277305446 |
| **PC 16:0;0_20:3;0** | 0.100260839044679 | -0.0337539196933179 | 0.007363170164889 | 0.0845228527102231 | -0.039347862157988 |
| **PC 16:0;0_20:4;0** | 0.0885253919534686 | 0.0051626139820744 | -0.103730904109545 | 0.0443792508575134 | 0.076249295877577 |
| **PC 16:0;0_20:5;0** | 0.0453585868853578 | -0.00917834326165006 | -0.178851308027161 | 0.0696325805932832 | 0.0485666773465181 |
| **PC 16:0;0_22:4;0** | 0.0850436350834202 | 0.00221150491005932 | -0.00348470951054199 | 0.0775358092195861 | 0.0473315428966455 |
| **PC 16:0;0_22:5;0** | 0.0984387782858399 | -0.00241865141404746 | -0.127092148986077 | 0.0650020988476731 | 0.067403226168303 |
| **PC 16:0;0_22:6;0** | 0.0720235082532455 | 0.005379579388219 | -0.184388058989567 | -0.0251487678229793 | 0.0467519261717216 |
| **PC 16:1;0_18:0;0** | 0.0657061026120251 | -0.0136162569838785 | -0.00511964894067503 | 0.153897209857828 | -0.0196155786759121 |
| **PC 16:1;0_18:1;0** | 0.0929489225571512 | 0.0335643682362013 | 0.0346570492826083 | 0.0618381386464522 | -0.0490059144126367 |
| **PC 16:1;0_18:2;0** | 0.0867383609016664 | 0.0521392968237658 | 0.119049077886738 | -0.0368117334597566 | -0.0990939388463473 |
| **PC 16:1;0_20:4;0** | 0.0768794139511156 | 0.0423523722985944 | -0.0495453416197918 | 0.0185057737845508 | 0.0429390521809048 |
| **PC 17:0;0_18:2;0** | 0.0951365975353527 | 0.0616434316817871 | 0.0528698886250514 | -0.0600504302981291 | -0.0762128753310395 |
| **PC 17:0;0_20:3;0** | 0.0840738579035499 | 0.0094842884691944 | 0.0315147817442503 | 0.0224347347261294 | -0.0450492372842659 |
| **PC 17:0;0_20:4;0** | 0.0950233113028248 | 0.0287791087482054 | -0.0633859926487503 | -0.0134569593952366 | 0.0529970984688507 |
| **PC 18:0;0_18:1;0** | 0.099265172150854 | 0.0206968500513502 | -0.00919058445362475 | 0.133424501818263 | -0.0256273705662721 |
| **PC 18:0;0_18:2;0** | 0.109142680292766 | 0.062750279489067 | 0.0665385834438364 | -0.0581686666793199 | -0.0730348845852701 |
| **PC 18:0;0_18:3;0** | 0.0862634789603388 | 0.00902583968732816 | -0.0420298444448721 | 0.11425241004362 | -0.041316202560527 |
| **PC 18:0;0_20:2;0** | 0.090053833298966 | 0.0169711135229898 | 0.012181291838221 | 0.0222720568269106 | -0.0591089658014436 |
| **PC 18:0;0_20:3;0** | 0.0959210250047208 | -0.0418888646438782 | -0.00781458867956199 | 0.0477651702904576 | -0.0432894876180045 |
| **PC 18:0;0_20:4;0** | 0.0923339044703348 | 0.0142094041104171 | -0.110430236590901 | -0.041810453048386 | 0.077286988873182 |
| **PC 18:0;0_20:5;0** | 0.0482908218622857 | -0.00573259715609674 | -0.17606978496749 | 0.0447200965852695 | 0.0479787936535158 |
| **PC 18:0;0_22:5;0** | 0.0989824492309784 | -0.0180638191764727 | -0.0710488045641367 | 0.0739548662490777 | 0.0272199948908691 |
| **PC 18:0;0_22:6;0** | 0.0796404681818682 | -0.00986075744226919 | -0.174872202235802 | -0.0195855741992598 | 0.0284999255689874 |
| **PC 18:1;0_18:1;0** | 0.0864437588750315 | 0.0731103607545835 | 0.0829649216207519 | 0.00339921982776264 | -0.0398541708898557 |
| **PC 18:1;0_18:2;0** | 0.0876148315582285 | 0.0819244563874299 | 0.143298591014656 | -0.0672963808053721 | -0.0734351319587977 |
| **PC 18:1;0_18:3;0** | 0.0656542286384046 | 0.0417927769255916 | 0.0190173341881439 | 0.0416484423774136 | -0.00203344054060999 |
| **PC 18:1;0_20:2;0** | 0.0758503650474752 | 0.0212922020481314 | 0.0843311056725292 | 0.023225891876738 | -0.0740725317169318 |
| **PC 18:1;0_20:3;0** | 0.0974507186578307 | 0.00520944913225199 | 0.0497202432202804 | -0.0115200057151062 | -0.026230621641659 |
| **PC 18:1;0_20:4;0** | 0.0915378546323356 | 0.0426248646135696 | -0.0302780154073063 | -0.0364184147214454 | 0.0678331363423938 |
| **PC 18:2;0_18:2;0** | 0.0791405873507484 | 0.0862393806530956 | 0.153598612335153 | -0.0443861891243895 | -0.0217992331351018 |
| **PC 18:2;0_20:3;0** | 0.0773191017080877 | 0.0288555870706803 | 0.0997270274587062 | 0.0415247395776424 | -0.018552206604158 |
| **PC 18:2;0_20:4;0** | 0.0948796791950753 | 0.0655533303448107 | 0.0304540102721883 | -0.0221210541801317 | 0.052266804535835 |
| **PC O-16:0;0/16:0;0** | 0.0469652433169796 | 0.0281050358728978 | 0.0372995282587761 | 0.0271785908029952 | 0.192010956287536 |
| **PC O-16:0;0/16:1;0** | 0.0456487307165529 | 0.0277423604777957 | 0.00137570751172953 | 0.0910978579932633 | 0.0124591318549209 |
| **PC O-16:0;0/18:1;0** | 0.0765448641705693 | 0.114843032825296 | 0.0413177900183494 | -0.0270943083838179 | 0.0285037309831262 |
| **PC O-16:0;0/18:2;0** | 0.0656514715412271 | 0.124543893240715 | 0.066534763832287 | -0.0601052952637922 | 0.0287105749122304 |
| **PC O-16:0;0/20:3;0** | 0.0757784809502375 | 0.0474143489382962 | 0.0121089057947703 | 0.00870508124128947 | 0.000347382089286563 |
| **PC O-16:0;0/20:4;0** | 0.0643276637791245 | 0.0584329868878029 | -0.0658382321122453 | -0.0422553775786791 | 0.114958613906023 |
| **PC O-16:1;0/16:0;0** | 0.051251755888944 | 0.073339718845463 | 0.0155196336077993 | -0.00820988937564198 | 0.162606768958758 |
| **PC O-16:1;0/18:0;0** | 0.000316400133287834 | 0.0573780658476403 | 0.0751954491717448 | -0.0623104622718301 | 0.128745576621443 |
| **PC O-16:1;0/18:1;0** | 0.0495152826669918 | 0.138129817717072 | 0.00370644749627247 | -0.0062183748180571 | 0.0627574384484001 |
| **PC O-16:1;0/18:2;0** | 0.0710310386420288 | 0.130468179357106 | 0.0282524389927153 | -0.0425885113656375 | 0.00619932188093359 |
| **PC O-16:1;0/20:3;0** | 0.0539167143187913 | 0.0548260518972502 | -0.015822356463011 | 0.0064349925428858 | 0.0204613275578628 |
| **PC O-16:1;0/20:4;0** | 0.0672136969279964 | 0.0890984794510342 | -0.0835933440687823 | -0.0546876810043309 | 0.102416827499896 |
| **PC O-16:2;0/18:0;0** | 0.0287070638941375 | 0.0530238999500148 | 0.0842521961746637 | -0.00333020749262243 | 0.139310500282543 |
| **PC O-17:0;0/15:0;0** | 0.0575172895590256 | 0.0330981158984562 | 0.00613763791688274 | 0.101958044252784 | -0.011831205286304 |
| **PC O-17:0;0/17:1;0** | 0.0696675597469232 | 0.0619965205751677 | 0.00284578870167836 | 0.102333564080922 | -0.0470601773666428 |
| **PC O-18:0;0/14:0;0** | 0.0174525601670206 | 0.0137146527119556 | 0.0617494455656104 | 0.00555038217589134 | 0.182298862188503 |
| **PC O-18:0;0/16:1;0** | 0.0221208913969282 | 0.0529796205599669 | 0.0045504436252924 | 0.0130389702281706 | 0.00356851768713114 |
| **PC O-18:0;0/20:4;0** | 0.0660734481382202 | 0.0912022471081077 | -0.0121221106851413 | -0.0796984142143287 | 0.0476710043238156 |
| **PC O-18:1;0/16:0;0** | 0.0657050034847866 | 0.118475661604101 | 0.0399083872202468 | -0.0666338095722992 | 0.0101724568818908 |
| **PC O-18:1;0/18:2;0** | 0.0584358168297192 | 0.127298650752162 | 0.0845703945684189 | -0.0809470360764093 | -0.0184871608724089 |
| **PC O-18:1;0/20:3;0** | 0.0631420532195644 | 0.0617743733028891 | 0.0781995901645427 | -0.0136984674324963 | -0.00703250507132158 |
| **PC O-18:1;0/20:4;0** | 0.0711854351226129 | 0.0941008282720947 | 0.00497253560091769 | -0.104063282207539 | 0.0723706450789618 |
| **PC O-18:2;0/16:0;0** | 0.0710336566240059 | 0.118638871805031 | 0.0533089971361933 | -0.0455073796277838 | 0.0172858523896498 |
| **PC O-18:2;0/18:1;0** | 0.048536625736409 | 0.108440031483602 | 0.056340519423378 | -0.0659150571936116 | -0.0182753079105294 |
| **PC O-18:2;0/18:2;0** | 0.0650052394019414 | 0.121838259893223 | 0.0790060266214678 | -0.0996683627288095 | -0.00719349921588155 |
| **PC O-18:2;0/20:4;0** | 0.055889003470278 | 0.0722527560020346 | -0.0338774495749373 | -0.0824545490331774 | 0.0290676891278975 |
| **PE 16:0;0_18:2;0** | 0.0707193629618388 | -0.0255659911121375 | 0.100335800810907 | 0.0555823307222451 | -0.0664248618470135 |
| **PE 16:0;0_20:4;0** | 0.0748374426140656 | -0.0294560464166978 | 0.0342649258306806 | 0.0764093362456039 | -0.014629559990274 |
| **PE 18:0;0_18:2;0** | 0.0855253935438937 | -0.0549050474573901 | 0.117936831408335 | 0.02246140389491 | -0.0723192875853222 |
| **PE 18:0;0_20:4;0** | 0.0883066489207552 | -0.0459322765944862 | 0.0247720157446215 | 0.00954602878812154 | -0.00416930573934403 |
| **PE 18:1;0_18:1;0** | 0.0522573882375261 | -0.0145468595249567 | 0.10155307115966 | 0.0267131501788868 | -0.0185875779227936 |
| **PE O-16:1;0/18:2;0** | 0.0457439403591785 | 0.053772336477166 | 0.0527602377510206 | -0.0358469350054453 | -0.0295223499892008 |
| **PE O-16:1;0/20:4;0** | 0.0633067239913863 | 0.052018455605853 | -0.0461744152888448 | -0.0379331822531344 | 0.0731422158046871 |
| **PE O-18:1;0/18:2;0** | 0.0618104420107843 | 0.101478828527679 | 0.106061526899783 | -0.0489433921575647 | -0.0201237387165424 |
| **PE O-18:1;0/20:4;0** | 0.0673634858118861 | 0.0687413820698013 | -0.0368963677267603 | -0.0492489274797861 | 0.076610944321887 |
| **PE O-18:2;0/18:1;0** | 0.0508924887285822 | 0.0857746443494163 | 0.0479785842458446 | -0.0286378961767973 | 0.00116320003492461 |
| **PE O-18:2;0/18:2;0** | 0.0638724931578676 | 0.0871521023666076 | 0.085624807791119 | -0.0722861120147332 | 0.0165900876343454 |
| **PE O-18:2;0/20:4;0** | 0.0718263042543885 | 0.0697517332527873 | -0.0549897257254597 | -0.0594578526717596 | 0.0745754084554747 |
| **PI 16:0;0_18:1;0** | 0.071155053654878 | 0.0126744710180461 | 0.0465884958499584 | 0.089477556857287 | -0.0519355879020551 |
| **PI 16:0;0_18:2;0** | 0.0798990926551281 | -0.0231010087959662 | 0.062879370379061 | 0.044844563308651 | -0.0612607571408367 |
| **PI 16:0;0_20:4;0** | 0.0666186448174527 | -0.0414817147585391 | -0.033573151242695 | 0.116505660831094 | 0.00763414431020687 |
| **PI 18:0;0_18:1;0** | 0.0901247702528812 | 0.022362707746495 | 0.0488678303297737 | 0.0833646653141123 | -0.0317850154632715 |
| **PI 18:0;0_18:2;0** | 0.0872847510180621 | 0.0030379138522421 | 0.0841353861197925 | -0.0237224850500233 | -0.0579426740620678 |
| **PI 18:0;0_20:3;0** | 0.088580556782916 | -0.0252564518672539 | 0.0703686203872627 | 0.0485821112352989 | -0.0643668158002973 |
| **PI 18:0;0_20:4;0** | 0.106161047037697 | -0.00792643944114998 | -0.0331774407790341 | -0.0329787161372701 | 0.0297678343664236 |
| **PI 18:1;0_18:1;0** | 0.0738135800635515 | 0.0590121958913759 | 0.105693217659789 | 0.00859153850611322 | -0.0286617245335158 |
| **PI 18:1;0_18:2;0** | 0.0568529114915406 | 0.0355869803278964 | 0.12219986745785 | -0.0671283335870923 | -0.0140483868438193 |
| **PI 18:1;0_20:4;0** | 0.0898161561218385 | 0.0062425755834194 | 0.0562731883203108 | 0.00867810520498256 | -0.00131431108738806 |
| **PI 18:2;0_18:2;0** | 0.0104086825421034 | 0.0426921211671191 | 0.0588176205279049 | -0.0542630576853592 | 0.00014347836905739 |
| **SM 32:1;2** | 0.0908081028329786 | 0.0607624770063416 | -0.0601524449373766 | 0.020641639296219 | -0.0935179212580004 |
| **SM 34:0;2** | 0.0580820925793329 | 0.0539191478758979 | -0.063611422309947 | -0.0213451446764802 | -0.0489447589092957 |
| **SM 34:1;2** | 0.0886863693468683 | 0.099097598251369 | -0.0766203779155969 | -0.0672750637873724 | -0.061323512621013 |
| **SM 34:2;2** | 0.090793919499569 | 0.0836097169055206 | -0.0660559697963932 | -0.0737263800233788 | -0.0569736914415256 |
| **SM 36:1;2** | 0.0766963486250738 | 0.052657746215839 | -0.125123857896613 | -0.0585776119326726 | -0.0817027715718572 |
| **SM 36:2;2** | 0.0733987587339203 | 0.0670691834512791 | -0.0997633046976369 | -0.0947110149124262 | -0.0632197263932092 |
| **SM 38:1;2** | 0.0826670339265817 | 0.0484945560255817 | -0.0735604547224991 | -0.0600462033976197 | -0.118721185391227 |
| **SM 38:2;2** | 0.0704774899104467 | 0.078530483449354 | -0.0635178210301681 | -0.0987506912182847 | -0.0993575574701056 |
| **SM 40:1;2** | 0.0811766089400087 | 0.0439712568903065 | -0.0616796321823788 | -0.0767983614180354 | -0.099714654320438 |
| **SM 40:2;2** | 0.0770657893665417 | 0.0780710983124021 | -0.0779043515624674 | -0.0833983850781587 | -0.102337461850101 |
| **SM 42:2;2** | 0.068714558815339 | 0.0658261950086843 | -0.14433101400497 | -0.0524124810937627 | -0.0510097126485249 |
| **TAG 46:1;0** | 0.046152898269886 | -0.0946474298250436 | 0.0360445285684371 | 0.100732772506671 | -0.0206868612625522 |
| **TAG 46:2;0** | 0.0469372941102148 | -0.0896679828052441 | 0.0448019054881609 | 0.0799442511769026 | -0.0147846411516576 |
| **TAG 48:0;0** | 0.0364160010694825 | -0.0856284741070734 | 0.017396263711453 | 0.0954995081514325 | -0.0061549316218997 |
| **TAG 48:1;0** | 0.0560427661275718 | -0.118070328108468 | 0.0265178010900065 | 0.0971516307486087 | -0.0187050891968708 |
| **TAG 48:2;0** | 0.0656347624155909 | -0.126326922241353 | 0.0422423810134372 | 0.076971027615347 | -0.0227294909879217 |
| **TAG 48:3;0** | 0.0660939277246232 | -0.122736060560396 | 0.0572736520315365 | 0.0312731075710165 | -0.0193259515365209 |
| **TAG 49:1;0** | 0.055714827813481 | -0.103004255895353 | 0.0218930974744906 | 0.0798980785506811 | -0.0304656424330035 |
| **TAG 49:2;0** | 0.0574546890850883 | -0.100803854913177 | 0.0205056284065126 | 0.0486925972198015 | -0.02727543315269 |
| **TAG 50:1;0** | 0.0579940706119712 | -0.127100071649717 | 0.0223239996045779 | 0.0708196977415431 | -0.00807348490504252 |
| **TAG 50:2;0** | 0.0719019054345145 | -0.139290675018803 | 0.0265913824949581 | 0.0438633671345615 | -0.01357917349126 |
| **TAG 50:3;0** | 0.0744158557514613 | -0.142345138991786 | 0.0383787456834304 | -0.0200902577458101 | -0.0145470957300017 |
| **TAG 50:4;0** | 0.0750309839652248 | -0.139289120679784 | 0.0458026680501869 | -0.0407129240528416 | -0.0116783345760906 |
| **TAG 50:5;0** | 0.0720000506814733 | -0.127249251137485 | 0.0232641468528754 | -0.00772160875781676 | -0.00126731071060008 |
| **TAG 51:1;0** | 0.0594122305050734 | -0.114256112813063 | 0.0178908867019301 | 0.0683526208567054 | -0.0220207660356683 |
| **TAG 51:2;0** | 0.0743682463689646 | -0.132041569627376 | 0.0243554294163089 | 0.0192534333118185 | -0.0251469757724247 |
| **TAG 51:3;0** | 0.075327262604775 | -0.133124345293476 | 0.0319817314407753 | -0.0551122048441221 | -0.0220321464261823 |
| **TAG 51:4;0** | 0.0718041581323617 | -0.118312291405288 | 0.025598684335172 | -0.0770538877684141 | -0.0142765047282537 |
| **TAG 52:2;0** | 0.0705266674436684 | -0.131736648067077 | 0.0283892547081196 | -0.0265632920868862 | -0.000172619357574438 |
| **TAG 52:3;0** | 0.0699483520766118 | -0.134623051654766 | 0.0399690851871775 | -0.117080701601509 | -0.00161483475892977 |
| **TAG 52:4;0** | 0.064532126515051 | -0.127832474956404 | 0.0451618267792186 | -0.149757298430845 | -0.00152344965258941 |
| **TAG 52:5;0** | 0.0683838585790526 | -0.131416681420094 | 0.0312377920078621 | -0.1179958418782 | 0.00363223579066133 |
| **TAG 52:6;0** | 0.0691039497969735 | -0.133820386263376 | -0.0312622797961059 | -0.0409221168206247 | 0.0197138837916523 |
| **TAG 53:2;0** | 0.0763787412025897 | -0.123256885482225 | 0.0321565094377506 | -0.0154973500554116 | -0.00905234277943604 |
| **TAG 53:3;0** | 0.0768796216967841 | -0.124264194965205 | 0.0394900329956563 | -0.0948129983874956 | -0.00638065286923612 |
| **TAG 53:4;0** | 0.0756989574617331 | -0.123880427366491 | 0.0389632983033964 | -0.131611253964578 | 0.00117617490731747 |
| **TAG 54:3;0** | 0.0686610081153134 | -0.113296766313381 | 0.0665297027349234 | -0.10479783785244 | 0.0115193953446231 |
| **TAG 54:4;0** | 0.0635681333458126 | -0.108380339316736 | 0.0744736260277086 | -0.162927428413258 | 0.0193083081824736 |
| **TAG 54:5;0** | 0.0641349288633011 | -0.114935152371712 | 0.0541386245167882 | -0.149910714295598 | 0.0277834460623663 |
| **TAG 54:6;0** | 0.0644574327293035 | -0.131153380550772 | -0.0199978539577196 | -0.110586828771398 | 0.0344279964085398 |
| **TAG 54:7;0** | 0.0575179059933658 | -0.120636956038396 | -0.0858580773732729 | -0.0655324656139006 | 0.0367260927011678 |
| **TAG 56:3;0** | 0.0430208049805026 | -0.0667992588160181 | 0.0334904305461957 | -0.0380785557930417 | 0.011025406060339 |
| **TAG 56:4;0** | 0.0642911055421782 | -0.0988699617113579 | 0.0634973788894773 | -0.0813120369193923 | 0.0131558517143267 |
| **TAG 56:5;0** | 0.074503207754955 | -0.107441130387706 | 0.00183217905110397 | -0.0991327879742594 | 0.0300446214286862 |
| **TAG 56:6;0** | 0.0773363205790844 | -0.123780355585326 | -0.036351038910699 | -0.0937467844261243 | 0.0535108792488694 |
| **TAG 56:7;0** | 0.0543419758416242 | -0.111740748635766 | -0.118875708755035 | -0.0726977175179541 | 0.0423473496036547 |
| **TAG 56:8;0** | 0.0436239577831177 | -0.0951379090045236 | -0.120715539198982 | -0.0955559266236681 | 0.034792631487954 |
| **TAG 58:7;0** | 0.0618295916000348 | -0.110889466291979 | -0.0902554840400024 | -0.0505566350407451 | 0.0416516483015111 |
| **TAG 58:8;0** | 0.0420053893206607 | -0.0762581279742669 | -0.136598960997408 | -0.0784075663459243 | 0.0394536667983388 |
| **TAG 58:9;0** | 0.032539052149013 | -0.0670035130782487 | -0.127760987975957 | -0.0912527283389073 | 0.0476267914009623 |

**Table S9.** Loadings of protein PCs 1-5 (MHO vs NOC).

| **Proteins** | **PC1** | **PC2** | **PC3** | **PC4** | **PC5** |
| --- | --- | --- | --- | --- | --- |
| **X.O_171_CD69** | 0.07930765231381 | 0.19460197312284 | -0.0347198738308394 | -0.000226252071597151 | 0.00207170210585051 |
| **X.184_CASP8** | 0.0983002558832966 | 0.0783637715569413 | -0.0354968755712761 | -0.000333343882229464 | -0.0439219300972317 |
| **X.O_170_CASP3** | 0.0968038458930268 | 0.20427988207506 | -0.0239869798632398 | 0.00256505172163421 | -0.0405762221188636 |
| **X.141_GH** | 0.00169159330727251 | 0.00760868529337343 | 0.0215054887931788 | 0.00270974846438311 | 0.37565678190911 |
| **X.185_CTSL1** | 0.103246262548346 | -0.0931844739043774 | -0.0279948800909361 | 0.00298626687016046 | -0.0400287693010304 |
| **X.O_131_IL7** | 0.0978940123800228 | 0.183963640435817 | 0.0263747046413036 | -0.00392546972832953 | -0.0083699597002349 |
| **X.O_153_ErbB2HER2** | 0.086266491429895 | -0.0109127001889641 | 0.142122450692659 | -0.00430938481177301 | -0.167458242768651 |
| **X.131_FGF23** | 0.0837739362372913 | -0.0775293414469468 | -0.0437116242139528 | -0.00493752138133715 | 0.0624624382973804 |
| **X.O_137_FADD** | 0.0959134942255224 | 0.168839301989184 | 0.0402763572127223 | -0.00670569310456088 | -0.0422047585843566 |
| **X.109_EGF** | 0.100372722225428 | 0.171135963018883 | -0.101148939782853 | -0.00758796015423647 | 0.0252224867796467 |
| **X.160_IL16** | 0.107994534110974 | -0.0633744658421316 | -0.0328612933701482 | -0.0081668356249783 | -0.0219576183526383 |
| **X.110_OPG** | 0.103061881492524 | -0.0783051430076381 | -0.0356467174886537 | 0.00877999299923718 | 0.0482563009170597 |
| **X.115_MCP1** | 0.0956800591990184 | -0.0942510679099662 | -0.0720980770034322 | -0.0100316876962992 | 0.00687248999404572 |
| **X.O_160_CXCL5** | 0.0873134265327155 | 0.166201934267995 | -0.00848338092743141 | 0.0102223672128064 | 0.0766312298376165 |
| **X.144_FAS** | 0.108731010609493 | -0.0951660685819113 | -0.0599646378911824 | -0.0108064184668724 | -0.049225751998041 |
| **X.124_TNFR1** | 0.115566222412615 | -0.107812198669005 | -0.0161806903172004 | 0.0112240526782584 | 0.0144275124564983 |
| **X.107_PlGF** | 0.119342462822002 | -0.108155798695924 | -0.0428858329084644 | -0.0141274828240654 | -0.0515502821359833 |
| **X.192_NEMO** | 0.0695687038083569 | 0.157328267509429 | -0.066938268646395 | -0.0141543132571137 | -0.0331880067884461 |
| **X.176_CD40** | 0.103255412448616 | 0.103323104670759 | -0.082880124177607 | -0.0149648126940017 | 0.0254621553109988 |
| **X.O_174_CDKN1A** | 0.0846470476170256 | 0.182923295558396 | -0.0211523849671294 | 0.0159197929727211 | -0.0584212567463002 |
| **X.O_187_eIF4B** | 0.083840527931819 | 0.194304417818309 | 0.0260753643807386 | -0.0164099542144115 | -0.0279217140116401 |
| **X.O_196_FRalpha** | 0.0886097734204275 | -0.00543620133855675 | 0.166122818191185 | -0.0185673891115093 | 0.0626302213408924 |
| **X.125_PDGFsubunitB** | 0.108175429623754 | 0.154196286368258 | -0.100190842042148 | -0.0188818209954673 | 0.0129652700554791 |
| **X.105_CD40L** | 0.0994690364281735 | 0.16258469774467 | -0.0965626433532023 | -0.0199311907908766 | 0.0229594183502472 |
| **X.169_ITGB1BP2** | 0.0971457511034758 | 0.182869809387797 | -0.0869383477976298 | -0.0216525692994268 | 0.0408419699644614 |
| **X.O_186_THPO** | 0.0959786522258159 | 0.0938696593613365 | 0.115225901142765 | -0.0239042598108381 | 0.0180135618383692 |
| **X.O_166_LYN** | 0.0830944607767402 | 0.196752013251891 | 0.0244505527232286 | -0.0241500881020534 | -0.0400583559200352 |
| **X.O_150_CAIX** | 0.0435667005591942 | -0.0274263666602415 | 0.0995384192374641 | 0.0244111994325692 | 0.044095507866237 |
| **X.127_CSF1** | 0.110551272819958 | -0.0922134069529795 | -0.0192568163324152 | -0.0247009982418592 | 0.098016578691086 |
| **X.195_NTproBNP** | 0.0294623215908711 | -0.0574077644118664 | 0.0102588653015946 | 0.0250102109447558 | 0.166660618816759 |
| **X.O_120_LAPTGFbeta1** | 0.11514153165278 | 0.134572203006285 | 0.0636098674497381 | 0.0266778554730955 | -0.0287110081944712 |
| **X.O_126_PARK7** | 0.0956671027638687 | 0.164978959992803 | 0.0307747988525501 | -0.0274512083765956 | -0.00378334941626782 |
| **X.O_159_MIA** | 0.0771300025499926 | 0.00775665202312395 | 0.154282256967154 | -0.0301782530961093 | -0.0569701674673575 |
| **X.170_CXCL16** | 0.108531635052543 | -0.0835870216173759 | -0.0834613817375389 | -0.031440205373993 | -0.0051800882955875 |
| **X.138_TNFSF14** | 0.11148288802662 | 0.116118200813566 | -0.0943223244070073 | 0.0318330178092592 | 0.0620402357181937 |
| **X.140_MPO** | 0.087038178938625 | -0.0639683521829968 | -0.0538368727909607 | 0.032299972421001 | 0.0486841941601329 |
| **X.O_154_ErbB3HER3** | 0.0856269562929634 | 0.0260310258620668 | 0.192525225998413 | -0.0323338043148541 | -0.0130695045669903 |
| **X.O_173_TRAP** | 0.0820736635792356 | 0.0174334426011231 | 0.131455535014393 | 0.0328017689042987 | -0.185160795514102 |
| **X.O_129_IL12** | 0.0588156520975228 | -0.0252790460705647 | 0.122961820292188 | 0.0329781678831998 | 0.0892296573877293 |
| **X.149_REN** | 0.0654808233850399 | -0.0382380876679746 | -0.0214002359479232 | 0.035216859946006 | -0.157016281355477 |
| **X.137_HSP27** | 0.0938598321799723 | 0.153353665674241 | -0.0867321809752965 | -0.036319828095213 | 0.0426292075176587 |
| **X.128_CXCL1** | 0.0849170810935164 | 0.128290887906809 | -0.0811579729255624 | -0.0371097993214994 | 0.062797195514915 |
| **X.O_161_MK** | 0.102542236453667 | 0.0798199347472602 | 0.084724714303143 | 0.037404717859051 | 0.0384393973452725 |
| **X.154_mAmP** | 0.0329216521279359 | -0.015553464054903 | -0.0435375486461867 | -0.037953127306616 | -0.0623435755028589 |
| **X.O_179_MICA** | 0.0597088155572292 | -0.00396337920767708 | 0.101953911034443 | 0.0381955439650611 | -0.0222725601258001 |
| **X.134_IL6RA** | 0.0936575521513007 | -0.063619585594455 | -0.0739352390670978 | -0.0397549344214376 | -0.0466297597170588 |
| **X.O_167_Flt3L** | 0.0747821657754921 | -0.0167733822844668 | 0.146317538500916 | 0.0400157215128252 | 0.0205885759742893 |
| **X.117_Gal3** | 0.103480649133404 | -0.0703083322436679 | -0.0546386606203945 | -0.0403187787174136 | 0.0708930707987271 |
| **X.O_177_AR** | 0.0617593259048139 | -0.0053007742934116 | 0.128827875684064 | 0.0406509308739049 | 0.0307469461464865 |
| **X.163_CCL4** | 0.0876901496828565 | -0.015756962173063 | -0.0443599901240684 | 0.040835084172442 | -0.0270209473690205 |
| **X.135_TNFR2** | 0.114118111851575 | -0.122872747190643 | -0.0365262483580964 | 0.0416379342587395 | 0.0260872079969697 |
| **X.161_MMP10** | 0.0590671079516372 | -0.0473721083220387 | 0.00279221519798619 | 0.0417662004166041 | 0.0813037191497789 |
| **X.172_SIRT2** | 0.101543179456835 | 0.183824378643834 | -0.097424311893399 | -0.0426708839147072 | 0.0348321664524614 |
| **X.O_152_EpCAM** | 0.0185514416166248 | 0.0131845880782305 | 0.0714749480164916 | -0.043326985707172 | 0.0917196551232045 |
| **X.191_CA125** | 0.0534610196738401 | -0.0318074700550879 | -0.0153826391323801 | -0.0440325797965016 | -0.0275154012480604 |
| **X.142_MMP1** | 0.0687010188721757 | 0.0940754487774121 | -0.0453048840281364 | 0.044247340655664 | 0.0580126918157742 |
| **X.183_SPON1** | 0.113319789381744 | -0.0893126368062147 | -0.0720091742033479 | -0.0451999071020904 | 0.0406210118008451 |
| **X.196_ECP** | 0.0609952417482101 | -0.0591689554625806 | -0.0370287549930632 | 0.0456813435973703 | 0.00502590102744458 |
| **X.168_CXCL6** | 0.101697969372091 | 0.110028661474001 | -0.0929991829555612 | -0.0461551309689467 | 0.0413217818306038 |
| **X.O_168_VEGFR2** | 0.081801813539238 | 0.0246035758934508 | 0.168657281496176 | -0.0463629263872738 | -0.0928792143746893 |
| **X.171_Dkk1** | 0.115804943786637 | 0.113777852890912 | -0.102845308102295 | -0.0476490917093124 | 0.0491048314087831 |
| **X.108_SELE** | 0.0848806482111295 | -0.0578214116137735 | -0.0517007889531224 | 0.0491153993552761 | -0.16042903882691 |
| **X.101_IL8** | 0.100357820880238 | 0.0119226139105348 | -0.0717511989383723 | 0.0509260368027105 | 0.0207360813064515 |
| **X.103_AM** | 0.0942615453608121 | -0.0999490316447974 | -0.0321456788732147 | 0.0529374996681317 | 0.0759489240206336 |
| **X.O_149_EMMPRIN** | 0.0802594432377181 | 0.0150326670630881 | 0.157751789649405 | -0.0542966591838757 | 0.0555511044803062 |
| **X.O_111_EZR** | 0.0822204684150422 | 0.040553255086017 | 0.174012621205309 | 0.0543298011437023 | -0.0643014049486887 |
| **X.133_IL18** | 0.0865462959608381 | -0.0615829200410381 | -0.0399482208157668 | 0.0546225523216403 | -0.0948515109206268 |
| **X.194_PECAM1** | 0.116780074516106 | 0.0527955900143793 | -0.0935801481838697 | -0.0557389169294285 | -0.067951070113922 |
| **X.143_RETN** | 0.0797732925603304 | -0.0613088477023633 | -0.048159535017557 | 0.0560006506707476 | 0.000646638476400256 |
| **X.O_176_TGFalpha** | 0.0851553483715533 | -0.0175744029584671 | 0.187261740373875 | 0.0574233805281294 | 0.0108851179376017 |
| **X.O_172_TNFRSF4** | 0.091074874827363 | -0.0284156430158281 | 0.15042947721505 | 0.0584971417332417 | 0.0525088228631368 |
| **X.O_128_CXCL11** | 0.0775382546033348 | 0.102027420024599 | 0.0275995580301708 | 0.0625087884663257 | 0.135692325603889 |
| **X.102_VEGFA** | 0.118814245586768 | 0.0128999082048239 | -0.0777089302969222 | 0.063329988317309 | 0.0239639461557888 |
| **X.120_TRAIL** | 0.102997795682968 | -0.080802523863105 | -0.0383182032429132 | -0.0645400005959859 | 0.00369320595433841 |
| **X.167_MMP7** | 0.0846362498859156 | -0.0274782456686634 | -0.0774735261880458 | 0.0653852840615657 | 0.0697861808175499 |
| **X.O_155_ErbB4HER4** | 0.0822106004664056 | 0.00610423286876948 | 0.176674042094323 | -0.0664883247192062 | -0.0250601153729843 |
| **X.193_FS** | 0.102732694068296 | -0.0385736736821907 | -0.0702486875569326 | 0.0667591315334915 | -0.0292039571609274 |
| **X.O_175_REG4** | 0.0775140242496055 | 0.0190487636763773 | 0.131074698332737 | 0.0676639556428824 | -0.0170539399680601 |
| **X.148_PTX3** | 0.0608283998710407 | -0.0645993676492526 | -0.0181729179499497 | -0.0689765794224498 | 0.0986719242679001 |
| **X.178_HBEGF** | 0.116125106850581 | 0.103664495348462 | -0.0819894150544775 | -0.0753243122218536 | 0.0618246498450394 |
| **X.157_PSGL1** | 0.0527270188000566 | -0.0339545144974958 | -0.018097407605711 | -0.0769646712233051 | 0.0507375486215219 |
| **X.155_TRANCE** | 0.0642889410955445 | -0.0559817301080404 | -0.0165338102563832 | -0.0796480495169632 | -0.0133173947970703 |
| **X.151_ST2** | 0.0735416557823471 | -0.0618163389302054 | -0.0497283088509448 | -0.0812901686010706 | -0.191667109487932 |
| **X.177_tPA** | 0.073392210368303 | -0.0215203404647201 | -0.0694642099375309 | 0.0813045922838342 | -0.183980628024592 |
| **X.O_110_ILT3** | 0.0772052240261311 | -0.0133542422424553 | 0.128963350382972 | 0.0816441304693561 | 0.0144369006373495 |
| **X.O_189_VIM** | 0.0554539176132382 | 0.0520133185596195 | 0.0615752627832176 | 0.0819572141205371 | -0.0196675494356013 |
| **X.166_CCL3** | 0.0901407207831848 | -0.0615502653396508 | -0.0415627608189131 | 0.0836053805827817 | 0.0012823060492647 |
| **X.O_185_ITGA1** | 0.062290852340824 | -0.00132549164734012 | 0.186441104112794 | -0.0856726578844978 | -0.0060884046975621 |
| **X.114_CSTB** | 0.119213757982079 | -0.0191696427970786 | -0.081239900368242 | 0.0883107319453025 | 0.0184228979432075 |
| **X.130_TRAILR2** | 0.0986879210352321 | -0.0928848907071978 | -0.0354417415102585 | 0.089267953692771 | 0.0280476577834765 |
| **X.O_184_EGFR** | 0.0792211127374802 | 0.0121699356909436 | 0.154048856212027 | -0.0900640942221429 | -0.0761796760042206 |
| **X.136_MMP3** | 0.0713014350696307 | -0.054582717448393 | -0.0691339863384695 | -0.0917407081130447 | -0.27326086513515 |
| **X.189_LEP** | 0.0215820692848097 | -0.0174311132074977 | 0.0286546849652507 | 0.0918408676196861 | 0.274377750157233 |
| **X.159_TM** | 0.117759930153359 | -0.0845496781549907 | -0.0198875805097888 | -0.0929591457136629 | -0.0389061898007643 |
| **X.126_IL27A** | 0.0724566245005302 | -0.0968926071224703 | -0.0183463758586176 | -0.0945776449844667 | 0.132137585155054 |
| **X.118_PAR1** | 0.11811591302048 | 0.0921555024218445 | -0.0786361908628859 | -0.0955980616477254 | 0.0498114241078107 |
| **X.158_MB** | 0.0792320889704595 | -0.08589330280138 | -0.0372640983515722 | -0.0974733943023077 | -0.141122399925153 |
| **X.139_PRL** | 0.0416148973795328 | -0.0386484436543438 | -0.0134846272431574 | -0.100758344566435 | 0.13369559686734 |
| **X.181_VEGFD** | 0.0652054957265405 | -0.0610959680779442 | 0.00452327224996993 | -0.100863615155849 | 0.166552789131512 |
| **X.152_TIM** | 0.0691961305939627 | -0.0564739084425478 | -0.0102629671579238 | 0.101551451929101 | -0.00758397965263689 |
| **X.145_PAPPA** | 0.0828628203607602 | -0.0378101850707499 | -0.0805032106764754 | -0.101643978928582 | -0.12934543995861 |
| **X.O_143_BAFF** | 0.0721071243898149 | -0.00295570761912611 | 0.142513034832505 | 0.102138054488896 | 0.0173945897304087 |
| **X.162_UPAR** | 0.106807435143479 | -0.0942457445117864 | -0.0253035919183122 | 0.10278272113973 | 0.102694219306814 |
| **X.111_SRC** | 0.00554219806478218 | -0.0253020662206663 | 0.0772068405460279 | -0.104243730624818 | 0.0841452382132612 |
| **X.121_hK11** | 0.0939684805767312 | -0.0917500189155837 | -0.00862897022918554 | -0.104468131107384 | 0.0327394699433721 |
| **X.187_FABP4** | 0.0722952007676353 | -0.0705973962883377 | -0.0285717000364275 | 0.105574276211199 | 0.147213432656644 |
| **X.122_TIE2** | 0.106152813350003 | -0.0848382092908301 | -0.0383589186733077 | -0.105823998518165 | -0.0409280457986981 |
| **X.150_CHI3L1** | 0.0731575653614837 | -0.0713875466472906 | -0.0395247208447793 | 0.107435254067354 | -0.0698755402573405 |
| **X.O_195_IL17RB** | 0.0493264863321195 | 0.00472763996418766 | 0.124777808489281 | -0.109688183975868 | -0.00458779905635166 |
| **X.156_HGF** | 0.114645587558107 | -0.0495003557007442 | -0.0538737913954653 | 0.109846741891405 | -0.0314105226728535 |
| **X.164_CTSD** | 0.109581723372606 | -0.041930857559797 | -0.0654291176758708 | 0.113893165275505 | -0.144601511295771 |
| **X.O_164_ICOSLG** | 0.06734507491539 | -0.00967611450752282 | 0.197293633684378 | -0.116082943596695 | -0.00622305918973322 |
| **X.O_165_CDH3** | 0.0642487943915186 | -0.0178275231786975 | 0.179273591310679 | -0.120832311745735 | -0.0148261671152056 |
| **X.O_151_CXCL10** | 0.060191818650137 | -0.0128787271400533 | 0.0916859800151181 | 0.123869932597243 | 0.0571298665036081 |
| **X.O_183_CXCL13** | 0.0734356527339349 | 0.0109122862867227 | 0.0844521683621372 | 0.125079296779415 | 0.0318516441317441 |
| **X.O_192_PRSS8** | 0.0810514836504756 | -0.021554756749388 | 0.0935490701668091 | 0.126554209835942 | -0.168921660734196 |
| **X.179_ESM1** | 0.089219905580244 | -0.0914980443063153 | -0.0580701522033211 | -0.131832722088787 | 0.0364807604225838 |
| **X.O_145_CCL19** | 0.0603937291389619 | -0.0116991790002973 | 0.0792626517456095 | 0.132307021716101 | 0.00957554726081951 |
| **X.129_LOX1** | 0.0691528446845807 | -0.0534236669197771 | -0.0170678212723079 | 0.132384387700176 | 0.0117223650406635 |
| **X.123_TF** | 0.0990474997212663 | -0.0947360477292708 | -0.0114648314410322 | -0.135860564901428 | 0.0453482709178908 |
| **X.O_182_HE4** | 0.0811884644224104 | -0.0373498037198784 | 0.116838720614112 | 0.138079186574625 | 0.0188683979722174 |
| **X.190_CCL20** | 0.0555793558866653 | -0.0404592703968595 | -0.0238345456635511 | 0.1381862488083 | 0.00687983829164489 |
| **X.174_AGRP** | 0.0944142644916983 | -0.0797182902373032 | -0.0215705216802285 | -0.139638873360748 | -0.0219786236696178 |
| **X.186_CX3CL1** | 0.0921011090005748 | -0.103642064960787 | -0.00948133975771755 | -0.141443509233719 | 0.113885507305798 |
| **X.O_133_CXCL9** | 0.0642410755220244 | -0.0194264426165078 | 0.0982300355403372 | 0.142707521095345 | 0.10166057946429 |
| **X.116_KLK6** | 0.0959045945859524 | -0.0877639136001275 | -0.0321666535728519 | -0.145743010742777 | -0.0257647567146865 |
| **X.O_142_FasL** | 0.0548749039664473 | -0.00711950474554097 | 0.153730890913798 | -0.150209867605631 | 0.0996204816252724 |
| **X.O_140_FUR** | 0.0920089743949103 | 0.0309427989649933 | 0.083502987408308 | 0.150546084403589 | -0.0760469590520836 |
| **X.165_RAGE** | 0.0788743828941588 | -0.082420350579431 | -0.0376603443459206 | -0.154018206909717 | 0.127130578601604 |
| **X.O_190_NTRK3** | 0.0751640997396834 | -0.00171868315802583 | 0.188983265392643 | -0.155967199970058 | -0.0431964643155748 |
| **X.106_GDF15** | 0.0991412205085676 | -0.0899951266371343 | -0.0597674197204083 | 0.165111935731503 | -0.0239349405350576 |
| **X.182_MMP12** | 0.0800110323911196 | -0.0604683187926733 | -0.0192178457462939 | 0.167237975925392 | 0.0318890703450765 |
| **X.112_IL1ra** | 0.0601302383055315 | -0.0544032098483742 | -0.0183103362472469 | 0.17750012077697 | 0.01238339518805 |
| **X.113_IL6** | 0.0579401960218792 | -0.0414472985070533 | -0.040355729484439 | 0.196123268876854 | -0.0148317507639377 |
| **X.173_GAL** | 0.0553549575007322 | -0.0454010114583672 | -0.0104588057879366 | -0.226332149181875 | -0.137925513238726 |
| **X.132_SCF** | 0.0580874059496323 | -0.0630479867197734 | 0.0133094628671506 | -0.238949796506917 | 0.055632836144716 |
